# Accuracy of mutational signature software on correlated signatures

**DOI:** 10.1038/s41598-021-04207-6

**Published:** 2022-01-10

**Authors:** Yang Wu, Ellora Hui Zhen Chua, Alvin Wei Tian Ng, Arnoud Boot, Steven G. Rozen

**Affiliations:** 1 Programme in Cancer and Stem Cell Biology, Duke-NUS Medical School, Singapore, 169857 Singapore; 2 Centre for Computational Biology, Duke-NUS Medical School, Singapore, 169857 Singapore; 3 Department of Biological Sciences, National University of Singapore, Singapore, 117558 Singapore

**Keywords:** Machine learning, Software

## Abstract

Mutational signatures are characteristic patterns of mutations generated by exogenous mutagens or by endogenous mutational processes. Mutational signatures are important for research into DNA damage and repair, aging, cancer biology, genetic toxicology, and epidemiology. Unsupervised learning can infer mutational signatures from the somatic mutations in large numbers of tumors, and separating correlated signatures is a notable challenge for this task. To investigate which methods can best meet this challenge, we assessed 18 computational methods for inferring mutational signatures on 20 synthetic data sets that incorporated varying degrees of correlated activity of two common mutational signatures. Performance varied widely, and four methods noticeably outperformed the others: hdp (based on hierarchical Dirichlet processes), SigProExtractor (based on multiple non-negative matrix factorizations over resampled data), TCSM (based on an approach used in document topic analysis), and mutSpec.NMF (also based on non-negative matrix factorization). The results underscored the complexities of mutational signature extraction, including the importance and difficulty of determining the correct number of signatures and the importance of hyperparameters. Our findings indicate directions for improvement of the software and show a need for care when interpreting results from any of these methods, including the need for assessing sensitivity of the results to input parameters.

## Introduction

**Figure 1:**
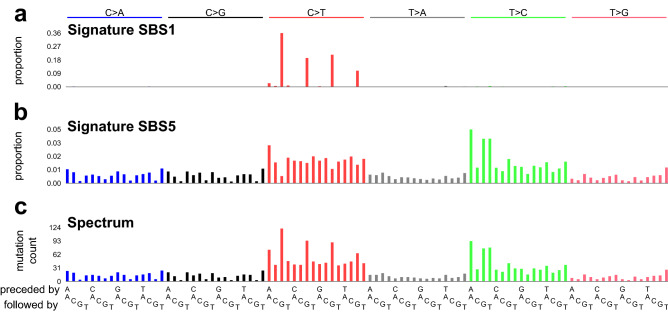
Mutational signatures and a mutational spectrum. (**a**) Mutational signature SBS1. Each vertical bar indicates the proportion of mutations of a particular mutation type—a single base mutation from a C or T in the context of its immediately preceding and following bases (seen at bottom of panel **c**). The height of each bar indicates the proportion of mutations of each type. The single base mutations are indicated on the top of the plot along with the color-code. For example, “C>T” indicates mutations from C to T and are represented by red bars. Mutations from A or G along with their preceding and following bases are reverse complemented. SBS1 is caused by deamination of 5-methylcytosine and consists almost entirely of CG-to-TG mutations. (**b**) Mutational signature SBS5, a relatively flat signature with unknown causes. (**c**) Example mutational spectrum composed of ~ 300 mutations from SBS1 and ~ 2000 mutations from SBS5. Each vertical bar indicates the number of mutations of a particular mutation type.

Mutational signatures are characteristic patterns of mutations generated by exogenous mutagens or endogenous mutational processes (Fig. 1). Here we focus on mutational signatures of single base substitutions in the context of the immediately preceding and following bases, which is by far the most-studied classification^1–3^. Examples of mutational signatures caused by mutagenic exposures include the signatures of tobacco smoke in lung cancer, of UV-exposure in skin cancer, and of aflatoxins in liver cancer^1,2,4,5^. Examples of signatures due to endogenous mutational processes include the signatures of deamination of 5-methylcytosine, of defective DNA mismatch repair, and of activated APOBEC cytosine deaminases^1,2,6–10^. Analysis of mutational signatures can (1) provide insights into mechanisms of DNA damage and repair^5–9,11^, (2) illuminate mutagenic processes associated with aging^12,13^, (3) reveal how mutagenic processes drive clonal expansion in normal tissue and set the stage for oncogenesis^13–19^, (4) suggest cancer prognoses and possible effective treatments^10,20^, and (5) lead to discovery of widespread mutagenic exposures that cause cancers^21^.

While mutational signatures can be delineated in experimental systems in which cells or animals are exposed to a mutagen and subsequently sequenced^4,22–24^, our focus here will be “signature extraction”, that is, the inference of mutational signatures by unsupervised machine learning from the somatic mutations in hundreds to tens of thousands of tumors^1,2,25^. Some methods for signature extraction have been assessed on simulated data and compared with one or two other methods^2,25–29^. However, to our knowledge, only 2 previous studies systematically assessed multiple methods^30,31^. Unlike these previous studies, here we restrict analysis to combinations of two signatures so that we can dissect in fine detail the determinants of accurate signature extraction. The Discussion examines the results of the current study in the context of the previous studies’ methods and findings.

One challenging aspect of mutational signature extraction is that the numbers of mutations attributable to two different signatures are sometimes positively correlated. Signatures SBS1 and SBS5 are notable examples (Fig. 1). We chose these two signatures for study because they stem from ubiquitous endogenous mutational processes and are detectable in a wide range of tumors and normal tissues^2^. Furthermore, their mutation loads positively correlate with patient age and sometimes also with each other^12^. Here, we assess the ability of 18 different computational methods to accurately extract (i.e., infer) mutational signatures from simulated data consisting of mixtures of these two signatures at varying proportions and correlations.

## Results

### Software tested

We considered 26 methods for signature extraction, and among these methods, found 18 suitable for testing^2,26–28,32–43^ (Supplementary Tables S1, S2). We excluded methods that did not use the most common classification of mutations as shown in Fig. 1, and Supplementary Table S2 details additional reasons for exclusion.

When running the software, we specified arguments and hyperparameters as suggested in the relevant publications and documentation, and if these were not available, default values. Supplementary Table S1 details the parameters selected and rationales for selecting them. Because most methods rely on random sampling, results often vary from run to run on the same input data. Therefore, excluding 2 methods with hard-coded, fixed random seeds, we ran each method 20 times on each data set, each time with a different, specified random seed.

### Synthetic data

We generated 20 sets of synthetic data, each consisting of 500 synthetic mutational spectra. The data sets had a range of values for two parameters:

- **SBS1:SBS5 ratio**, defined as the mean over the 500 spectra of (SBS1 mutation count) / (SBS5 mutation count). We generated data sets with SBS1:SBS5 ratios of 0.1, 0.5, 1, 2, and 10.
- **SBS1–SBS5 correlation**, defined as the Pearson ${R}^{2}$ of correlation between log_10_ of the number of mutations ascribed to SBS1 and log_10_ of the number of mutations ascribed to SBS5. We generated data sets with SBS1–SBS5 Correlations of 0.1, 0.2, 0.3 and 0.6.

There was one data set for each of the 20 possible combinations of values for the SBS1:SBS5 ratio and the SBS-SBS5 Correlation. The synthetic data sets are at https://doi.org/10.5281/zenodo.5510836.

### Evaluation measures

We assessed each method according to 4 measures:

- Cosine similarity to SBS1, the mean of the cosine similarities between SBS1 and each of the extracted signatures that are more similar to SBS1 than to SBS5, if any exist. Otherwise, if all signatures are more similar to SBS5 than to SBS1, then the cosine similarity between SBS1 and the extracted signature most similar to SBS1.
- Cosine similarity to SBS5, analogous to cosine similarity to SBS1.
- Positive Predictive Value (PPV), the number of true positives, $TP$*,* divided by the total number of extracted signatures. $TP$ is defined as follows: Let ${x}_{1}$ be the number of extracted signatures with cosine similarity to SBS1 > 0.9 and let ${x}_{5}$ be defined analogously for SBS5. Let ${c}_{1}$ be 1 if ${x}_{1}>0$, or 0 otherwise, and let ${c}_{5}$ be 1 if ${x}_{5}>0$ or 0 otherwise. Then $TP$ = ${c}_{1}+{c}_{5}$.
- True positive rate (TPR)*,* $TP$ divided by the number of ground-truth signatures, which is always 2.

To summarize the assessment of each method used a “Composite Measure”, defined as the sum of the 4 individual measures.

### Signature extraction when the number of signatures to extract was unspecified

We first evaluated signature extraction on each of the synthetic data sets without specifying the number of signatures to extract, which is the usual case in practice. As with much of unsupervised learning, determining the number of items to learn, in this case the number of signatures, is a central challenge. Ten methods provide functionality to select the number of signatures to extract and 5 methods specify algorithms for selecting the number of signatures, which we implemented (Supplementary Table S1). For three methods, mutSignatures, signature.tools.lib and SomaticSignatures.NMF, there is no implementation and no specified algorithm for choosing the number of extracted signatures, and we tested these only in a later part of this study (Supplementary Table S1).

**Figure 2:**
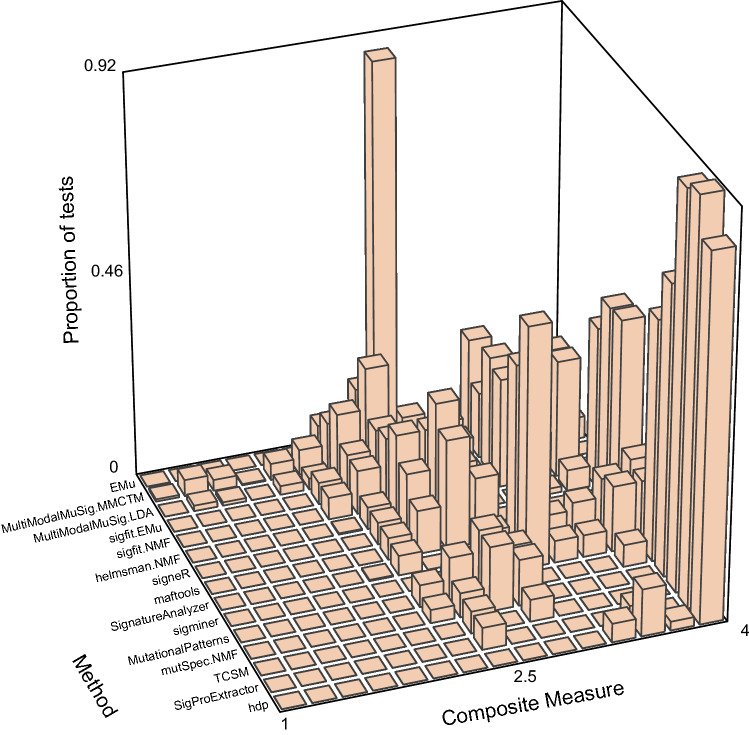
Composite measures of signature extraction results when the number of signatures was unspecified. The height of each bar is the proportion of all tests across all data sets and all random seeds. For most methods this comprises all 20 datasets with 20 random seeds per dataset (400 replicates total), while maftools and MutationalPatterns were run only once for each dataset because the random seed is not user modifiable. The methods are arranged from EMu to hdp by ascending mean Composite Measure.

**Figure 3:**
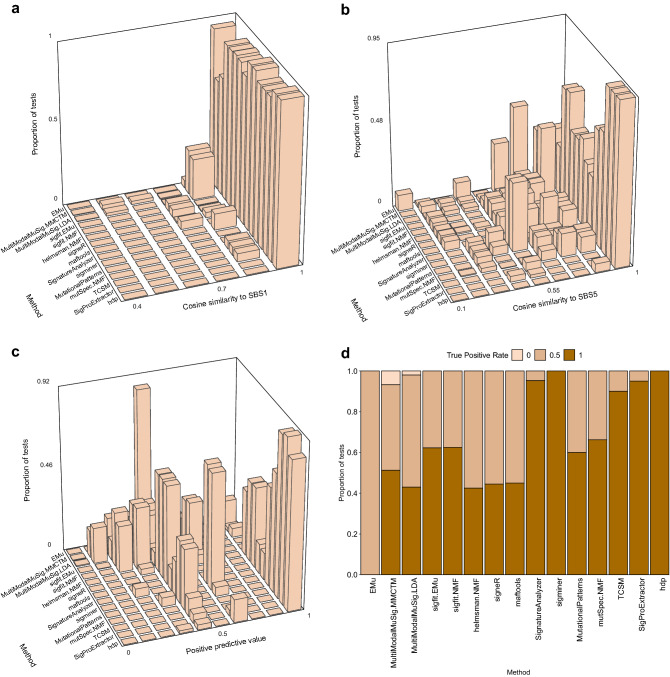
Individual measures of signature extraction performance when number of signatures was unspecified. The order of methods is as in Fig. 2. (**a**,**b**) The height of each bar indicates the proportion of all tests for a given method (on *y* axis) with the range of similarities to SBS1 or SBS5 indicated on the *x* axis. (**c**) The height of each bar indicates the proportion of all tests for a given method (on *y* axis) with the range of positive predictive values indicated on the *x* axis. (**d**) The height of each rectangle indicates the proportion of tests for a given method (*x* axis) with the true positive rate as indicated by the shade of the color in the rectangle. Because there are always 2 signatures in the synthetic data, the true positive rate takes only 3 values: 0 (none of the signatures detected), 0.5 (only one signature detected), 1 (both signatures detected).

Two topic-model based methods—hdp and TCSM—and two NMF-based methods—SigProExtractor and mutSpec.NMF—stood out as best able to extract the ground-truth signatures when the number of signatures to extract was not specified (Figs. 2, 3, Supplementary Tables S3, S4, full results at https://doi.org/10.5281/zenodo.5512002)^26,31,32,38^. These 4 methods usually extracted 2 signatures that were almost identical to SBS1 and SBS5 except at the most extreme SBS1:SBS5 Ratios (0.1 and 10) and the highest correlation (Supplementary Figs. S1–S4, Supplementary Tables S3, S4). They, as well as many other methods, usually extracted SBS1 more accurately than SBS5. This is consistent with our previous experience that sparse signatures such as SBS1, which consists almost entirely of only 4 mutation types, are more easily extracted than relatively flat signatures such as SBS5 (Fig. 1a,b)^2^. We also noted that the Composite Measures for SigProExtractor and TCSM were essentially identical across all the random seeds for each data set (Supplementary Figs. S5, S6). By contrast, the Composite Measures for mutSpec.NMF were extremely variable for different random seeds in many of the data sets, and this was also true for hdp in 2 data sets (Supplementary Figs. S7, S8).

**Figure 4:**
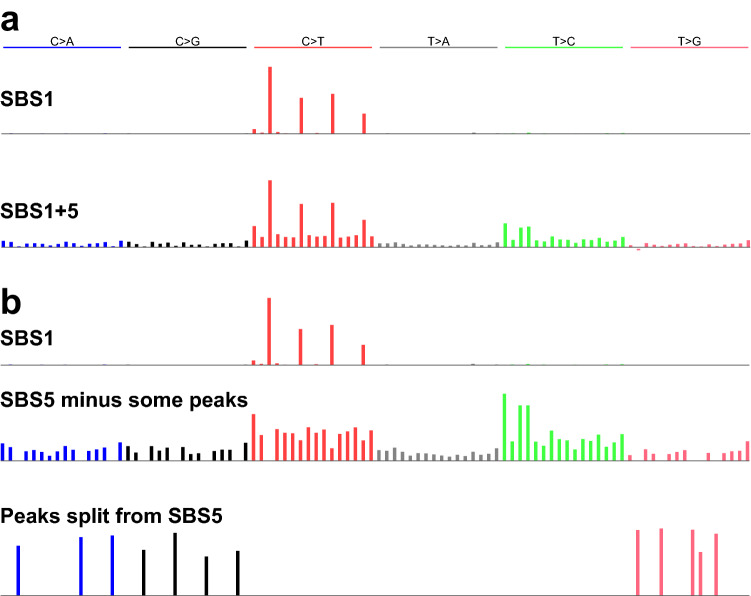
Two types of inaccurately extracted signatures. (**a**) Two signatures extracted by MutationalPatterns, consisting of an accurate version of SBS1 and a merge of SBS1 and SBS5 (“SBS1 + 5”). (**b**) Three signatures extracted by sigminer, consisting of an accurate version of SBS1, version of SBS5 lacking some peaks, and a third signature which contains the peaks lacking from the second signature.

Compared to the 4 most accurate methods, the other 4 methods—MutationalPatterns, maftools, signeR, and helmsman.NMF—had lower mean Composite Measures on data sets with SBS1:SBS5 Ratios ≥ 2 (Supplementary Figs. S9–S12). This was because, at these ratios, they did not extract SBS5, but rather a merge of SBS1 and SBS5 (denoted “SBS1 + 5”, Fig. 4a). EMu usually extracted 2 signatures: SBS1 and SBS1 + 5, but never extracted SBS5 regardless of the SBS1:SBS5 Ratio and correlation, and consequently had low TPRs (Supplementary Table S4).

**Figure 5:**
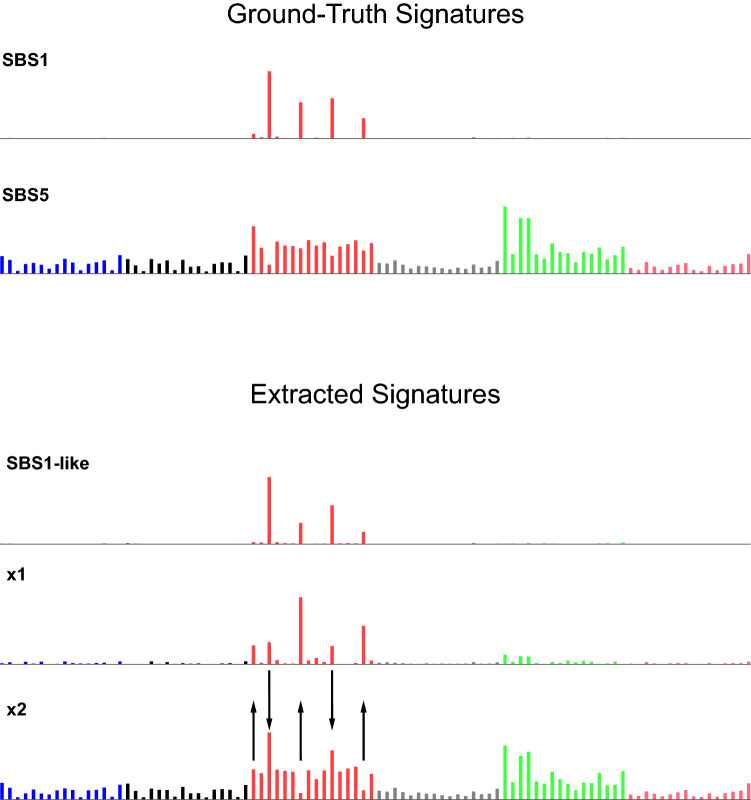
Examples of signatures resembling neither SBS1 nor SBS5. Above are signatures SBS1 and SBS5 for reference. Below are signatures extracted by MultiModalMuSig.MMCTM that comprise an SBS1-like signature and two signatures that do not closely resemble either SBS1 or SBS5. However, signature x1 somewhat resembles SBS1, with some mutations from 3 mutation types reallocated from SBS5 to x1 (up arrows). Signature x2 somewhat resembles SBS5, with some mutations from 2 mutation types reallocated from SBS1 to x2 (down arrows).

**Figure 6:**
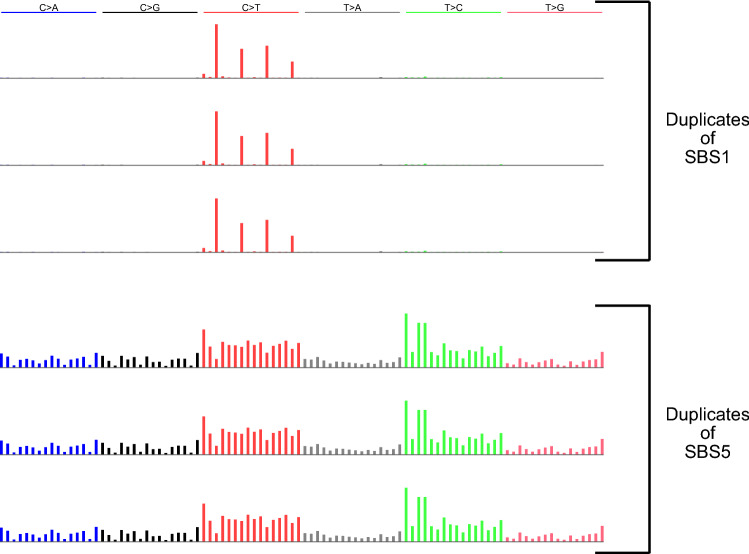
Examples of nearly identical duplicate signatures extracted by sigfit.NMF. Shown are 3 of the 4 duplicates of SBS1 and 3 of the 4 duplicates of SBS5 discovered by sigfit.NMF with seed = 1 in the data set with SBS1:SBS5 ratio = 1 and ${R}^{2}$ = 0.2.

The remaining methods usually extracted ≥ 3 signatures—as many as 4.54 signatures on average for sigfit.EMu—and consequently had low PPVs (Supplementary Table S3, S4). Two methods, sigminer, and SignatureAnalyzer, often extracted SBS1 and one or two split versions of SBS5, in which the peaks for some mutation types were put in a separate, extremely sparse, signature (Fig. 4b). They thus had low PPVs (Supplementary Table S4). MultiModalMuSig.LDA and MultiModalMuSig.MMCTM had highly variable results in each data set (Supplementary Figs. S13, S14). They also extracted signatures that included nearly identical duplicates of SBS1, SBS5, SBS1 + 5, as well as signatures that did not closely resemble either of SBS1 or SBS5 (Fig. 5). Two methods, sigfit.NMF and sigfit.EMu, extracted multiple, nearly indistinguishable versions of SBS1 and SBS5 (Fig. 6).

### Signature extraction when the number of signatures to extract was specified as 2

**Figure 7:**
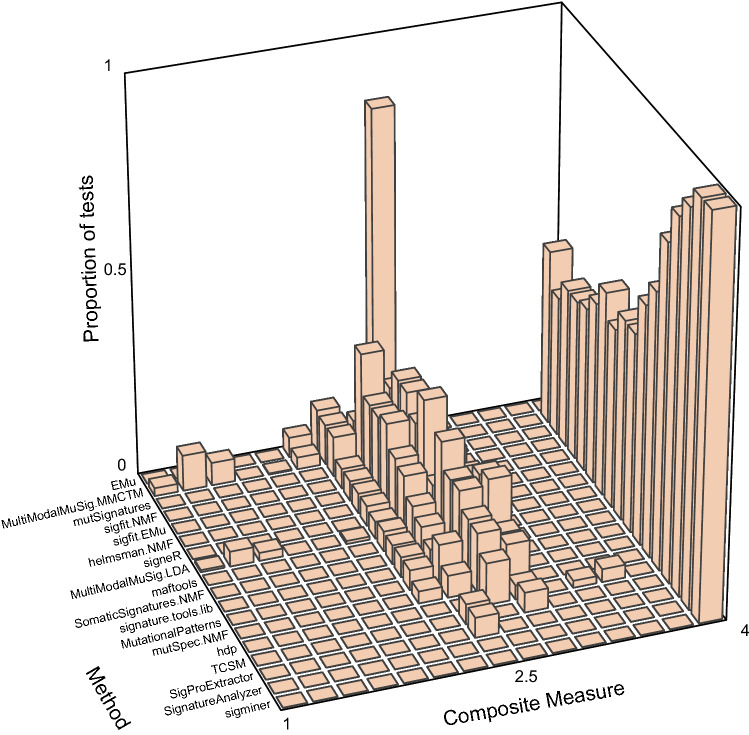
Composite Measure of signature extraction results when *K* was specified as or suggested to be 2. Methods are arranged by ascending mean composite measure.

When the number of ground-truth signatures was unspecified, 4 of the methods (hdp, SigProExtractor, TCSM, and mutSpec.NMF) had substantially higher Composite Measures than other methods, but it was not clear whether this was solely because of better estimation of the number of signatures or whether other factors contributed. Therefore, we evaluated signature extraction on the same 20 data sets, but this time specifying or suggesting 2 signatures (Fig. 7, Supplementary Fig. S15, full results at https://doi.org/10.5281/zenodo.5512018). The performance of sigminer and SignatureAnalyzer improved markedly, as they no longer split SBS5 into 2 signatures, as they had done previously (Fig. 4b, Supplementary Tables S5–S7). The performance of MultiModalMuSig.LDA also improved, because it less often extracted multiple versions of SBS1, which led to better PPV, although results were still variable from run to run on the same data set (Supplementary Fig. S13). The performance of hdp, which does not allow exact specification of the number of signatures to extract, declined slightly (Supplementary Fig. S8), because it less often accurately extracted SBS5 (Supplementary Table S5, cell AC9). The results of the other methods with the best performance when $K$, the number of signatures to extract, was unspecified (SigProExtractor, TCSM, mutSpec.NMF) changed very little when $K$ was specified as 2 (Supplementary Figs. S5, S6, S7, Supplementary Table S5). The remaining methods still did not extract SBS5 from data sets with SBS1:SBS5 ratios ≥ 2; instead, as when $K$ was not specified, they extracted the SBS1 + 5 merge (Supplementary Tables S5–S7).

### Variable results from 5 methods that use the same NMF implementation

A notable result from the analyses above was that five of the methods—mutSpec.NMF, MutationalPatterns, signature.tools.lib, maftools, and SomaticSignatures.NMF—performed differently even though they use the same implementation of the Brunet NMF algorithm^44^ in the R NMF package^45^ (https://github.com/renozao/NMF, Figs. 2, 3, 7, Supplementary Fig. S15, Supplementary Tables S3–S7). To understand the reasons for these performance differences, we examined how the five methods used this NMF implementation. Among these 5 methods, signature.tools.lib is unusual in that it calls the nmf function multiple times on resampled data, as described in Table 1. For the input data in the current study, this strategy did not improve the Composite Measure compared to the single calls to the nmf function used by mutSpec.NMF and MutationalPatterns (Fig. 7, Supplementary Fig. S15).

**Table 1:** Differences among 5 methods that use the "brunet" method in the R NMF package.

	mutSpec. NMF	MutationalPatterns	signature.tools.lib	SomaticSignatures.NMF	maftools
K* specified as 2					
nrun	200	200	**	1	1
Hard-coded random seed	N	Y	N	N	Y
Add "pseudocount"	N	Y	N	N	N
Mean Composite Measure	3.54	3.44	3.334	3.331	3.25
${\varvec{K}}$* unspecified			No algorithmic selection of $K$		
nrun for NMF::nmfEstimateRank() (for each $K$ in $K$ = 2..10)	50	10			10
nrun for the NMF::nmf() function once $K$ is selected (same as when $K$ is specified as 2)	200	200			1
Hard-coded random seed	N	Y			Y
Add "pseudocount"	N	Y			N
Mean Composite Measure	3.54	3.43			3.25
Mean number of extracted signatures	2	2.1			2

*$K$ denotes the number of signatures to extract.

**Signature.tools.lib resamples the input matrix 20 times, and for each resampling replicate, it calls the NMF::nmf function with nrun = 200. From among the 200 results of each of the nmf calls, it selects a few results that approximately minimize reconstruction error. It then clusters all the selected results and returns the medoids of two clusters as the extracted signatures.

Among the 4 methods other than signature.tools.lib, in the simpler case, when $K$ was specified as 2, there are 3 differences in how nmf is used (Table 1). First, the nrun argument to nmf is 200 in mutSpec.NMF and MutationalPatterns but 1 in maftools and SomaticSignatures.NMF. This argument specifies the number of matrix factorizations to be carried out, each starting at a different random initial state. The final return value is the factorization that generated the product with the lowest Kullback–Leibler divergence from the input matrix. Second, maftools and MutationalPatterns hard-code a random seed of 123,456. Third, for reasons we could not find explained, MutationalPatterns hard-codes the addition of a “pseudocount” of 10^–4^ to each cell of the input matrix. The 4 methods generate identical results when nmf is called in the same way (Supplementary Table S8).

For the 4 methods that use a single call to the nmf function, we dissected the reasons for the differences in mean Composite Measure for the case where $K$ was specified as 2 (Table 1):

- MutationalPatterns had lower mean Composite Measures than mutSpec.NMF, for two reasons (Supplementary Figure S16, Supplementary Table S9). First, addition of the “pseudocount” by MutationalPatterns reduced the Composite Measure. Second, the hard-coded random seed of 123,456 imposed by MutationalPatterns was an unlucky choice. We assessed the Composite Measure of MutationalPatterns once per data set based on this hard-coded random seed, while we assessed the mean Composite Measure of mutSpec.NMF over 20 different random seeds for each data set. The hard-coded seed of 123,456 used by MutationalPatterns yields a lower Composite Measure than the mean Composite Measure from the other 20 random seeds (Supplementary Figs. S16, S17, Supplementary Tables S9, S10). Indeed, 123,456 yields the fourth-lowest Composite Measure among 21 random seeds tested.
- SomaticSignatures.NMF had a lower mean Composite Measure than MutationalPatterns because MutationalPatterns used nrun = 200 while SomaticSignatures.NMF used nrun = 1 (Supplementary Figure S18, Supplementary Table S11).
- maftools had a lower mean Composite Measure than SomaticSignatures.NMF again because the Composite Measure after calling nmf with the fixed seed of 123,456 is lower than the average Composite Measure over 20 random seeds (Supplementary Fig. S19, Supplementary Table S12).

When $K$ is unspecified, nrun must also be specified as an argument to the nmfEstimateRank function, which selects $K$ (the number of signatures, called the “factorization rank” in the NMF package). After $K$ is selected, a possibly different value of nrun is supplied to the nmf function for a final factorization. Thus, if nmfEstimateRank estimates $K$ correctly as 2, nmf is simply called with nrun = 2. For each of the factorizations carried out by nmfEstimateRank, mutSpec.NMF uses nrun = 50, while MutationalPatterns and maftools use nrun = 10. (SomaticSignatures.NMF and signature.tools.lib do not call nmfEstimateRank and do not automate selection of $K$*.*) For MutationalPatterns, the mean Composite Measure is lower when $K$ is unspecified than when $K$ is specified as 2 (Table 1). This was because, when *K* was not specified, the function nmfEstimateRank selected *K* = 3 in some tests. This in turn was because of three differences in between how MutationalPatterns and mutSpec.NMF call nmfEstimateRank (Table 1, Supplementary Fig. S20, Supplementary Table S13). Although maftools also calls nmfEstimateRank with nrun = 10 and a single hard-coded seed, when run with no pseudocount, nmfEstimateRank always estimated *K* = 2.

## Discussion

We assessed 18 methods for extracting mutational signatures on 20 synthetic data sets constructed from mutational signatures SBS1 and SBS5 (Fig. 1). In these data sets, the number of mutations due to each signature and the correlations between the signatures varied. When the number of signatures to extract was not specified in advance, which is the usual situation in practice, 4 methods—hdp, SigProExtractor, TCSM, and mutSpec.NMF—most accurately extracted signatures (had the highest Composite Measures, Figs. 2,3, Supplementary Tables S3, S4). When the number of signatures was specified or suggested in advance to be 2, sigminer and SignatureAnalyzer also extracted both signatures accurately (Fig. 7, Supplementary Fig. S15, Supplementary Tables S5, S6).

This study focused on a specific question regarding separating mutations generated by two correlated signatures, which let us dissect the reasons for differences in accuracy. The results highlighted the challenges and importance of accurately estimating the number of signatures. However, even when the correct number of signatures was specified to the software, there was considerable variability in performance. The most accurate methods were surprisingly robust both to the SBS1:SBS5 Ratio and to SBS1-SBS5 Correlation. Indeed, their accuracy degraded only for data sets with the most extreme SBS1:SBS5 ratios (0.1 or 10) and the highest correlation (Supplementary Figs. S1–S4, Supplementary Table S4, S7). By contrast, many of the other methods did not extract SBS5 from data with an SBS1:SBS5 ratio ≥ 2 (Supplementary Figs. S9–S12), but rather extracted a merge of SBS1 and SBS5 that we call SBS1 + 5 (Fig. 4a). Two methods extracted multiple instances of almost identical signatures but failed to merge them (Fig. 6), and two methods had extremely variable results from run to run on each data set (Supplementary Figs. S13, S14). Results from 5 methods based on the same implementation of the Brunet algorithm varied substantially, in some methods due to an inadequate, hard-coded number of iterations (nrun = 1) in the Brunet algorithm (Table 1).

We are aware of only two previous studies that systematically assessed multiple methods on the same data sets^30,31^. One of these studies evaluated 7 computational methods for signature extraction^30^. This study was not designed to assess the critical aspect of whether methods were able to accurately estimate the number of signatures to extract, because it provided the correct number of signatures to the methods. The study used several measures to evaluate the methods’ results. One measure was reconstruction error, i.e. how well the input spectra could be reconstructed from the extracted signatures, which is rarely a question of interest, because, as acknowledged in the study, most methods extract signatures that yield good reconstructions. A second measure was the specificity of the extraction, defined as the number of COSMIC signatures correctly not detected divided by the number of COSMIC signatures absent from the input synthetic data. However, incorrectly extracting a known signature is rarely a problem, and therefore all methods did well by this measure. Finally, a third measure was sensitivity. This indeed is an issue in signature extraction because methods often fail to extract signatures. Unfortunately, the sensitivity results in this previous study shed little light on the results of the current study because, out of the 6 methods with the highest Composite Measures in the current study when $K$ was specified as 2, the previous study only analyzed one: a version of SignatureAnalyzer (termed “bayesNMF” in^30^). In addition, the previous study did not report any measures of variability of sensitivity across replicates.

A more extensive previous study evaluated 14 methods on 37 synthetic data sets in a paper presenting the implementation of SigProExtractor^31^. Two authors of the current study (YW and SGR) are also authors on this previous study. There were two major differences in approach compared to the current study. First, the previous study assessed the signature extraction methods on a wide range of synthetic data designed to mimic the signature exposures in tumors, while the current study was designed to allow detailed dissection of the behavior of methods in analyzing two correlated signatures. Second, SigProExtractor was optimized on the synthetic data presented in the study and outperformed the other methods, while the methods and parameters used in the current study were not optimized on the synthetic data. Of the 4 methods that had the highest Composite Measures in the current study when $K$ was unspecified, only SigProExtractor and mutSpec.NMF were tested in the SigProExtractor study. SignatureAnalyzer extracted fewer false positive in the SigProExtractor study than in the current study. Possibly this was because the data sets in the SigProExtractor study had many more signatures per input sample, and thus SignatureAnalyzer may have overestimated $K$ less often. An important conclusion from both the SigProExtractor study and the current study is the importance of assessing methods on synthetic data.

We draw some broader conclusions from the current study. First, there was substantial variability in the Composite Measure across the methods, and the behavior of some methods suggests that they were not extensively tested. Examples of this include the multiple, nearly identical duplicate signatures returned by sigfit.NMF and sigfit.EMu (Fig. 6), the variable results from MultiModalMuSig.LDA and MultiModalMuSig.MMCTM across different random seeds for the same data set (Supplementary Figs. S13, S14), and the lack of tuning of the nrun argument to the nmf function in the R NMF package by several methods (Table 1). This again points to the importance of testing on a range of data sets, including those presented here and those in^1,2^.

Second, the results underlined the importance of selecting the correct number of signatures. Notably, in the current study, SignatureAnalyzer and sigminer tended to extract too many signatures when the number of signatures was not specified, but they extracted highly accurate signatures when the correct number of signatures to extract was provided. The importance of determining the number of signatures was also evident in the SigProExtractor study^31^. For example, in that study, sigfit.NMF estimated a $K$ that was on average only 34.8% of the true number of signatures, and consequently the average true positive rate was 33.1%. This in turn shows the importance of human judgement regarding the number of signatures present when assessing results in the light of all available evidence.

Third, the methods with the best performance in the current study have multiple parameters, including parameters that govern the amount of sampling done that affect the results (the number of burn-in and Gibbs-sampling iterations or bootstrap replicates, Supplementary Table S1). TCSM and hdp also require additional parameters and hyperparameters. The importance of these parameters, over and above the critical question of estimating the number of signatures to extract, again implies that use of the software and interpretation of the results require considerable expertise and depend on human interpretation of results in the light of all available evidence.

Finally, the results of the current study lead to some recommendations for best practices in extraction of signatures: one should do multiple runs with different random seeds to determine stability, do multiple runs to test sensitivity to parameters and hyperparameters, and make use of available diagnostics, especially regarding selection of the number of signatures to extract.

## Methods

### Generating synthetic data

We generated one data set for each of the 20 possible combinations of values for the SBS1:SBS5 Ratio and the SBS-SBS5 Correlation, using the CreateSBS1SBS5CorrelatedSyntheticData function in the SynSigGen package (https://github.com/steverozen/SynSigGen). The synthetic data sets are available at 10.5281/zenodo.5510836.

We generated each synthetic data set as follows:

1. Designate the signature that will have the larger number of mutations as the “main signature” and the other signature as the “correlated signature”.

2. Repeat the following steps until the Pearson’s ${R}^{2}$ of correlation between the two signatures is within 0.01 of the desired SBS1-SBS5 Correlation:

2.1. Generate 500 exposures to the main signature from a log_10_-normal distribution with $\upmu$ = 2.5 and $\sigma$ as specified in Supplementary Table S14. It was necessary to select the value of $\sigma$ by trial and error to enable generation of data with the desired correlation. The $\mu$ of 2.5 represents a reasonable number of mutations ascribed to either SBS1 or SBS5 based on the numbers of mutations ascribed to them in Ref.^2^. Discard and regenerate any exposures with < 100 mutations.

2.2. For each of the exposures, $e$, generated in Step 2.1, generate exposure to the correlated signature by first drawing $r$ from a log_10_-normal distribution with $\mu ={\mathrm{log}}_{10}(e)$ and with a $\sigma$ selected by trial and error to enable the target correlation (Supplementary Table S14). Set the exposure to the correlated signature as $r\cdot$(SBS1:SBS5 Ratio)^-1^ if SBS1 is the main signature, or $r\cdot$(SBS1:SBS5 Ratio) if SBS5 is the main signature. Discard and regenerate any exposures with < 1 mutation.

3. To generate each spectrum from the exposures to SBS1 and SBS5, multiply the exposure times the respective signature, add the two products, and then round. The profiles of SBS1 and SBS5 were taken from https://www.synapse.org/#!Synapse:syn12025148 (Ref.^2^).

### Code for running signature extraction software

R package SynSigRun (https://github.com/WuyangFF95/SynSigRun) contains codes to run each method and R package SynSigEval (https://github.com/WuyangFF95/SynSigEval) has functions to evaluate the methods. Five methods rely on the R NMF package; we used version 0.30.1 (https://github.com/renozao/NMF).

## Supplementary Information

Supplementary Figures.

Supplementary Tables.

## References

[1] Alexandrov LB (2013). Signatures of mutational processes in human cancer. Nature.

[2] Alexandrov LB (2020). The repertoire of mutational signatures in human cancer. Nature.

[3] Poon SL, McPherson JR, Tan P, Teh BT, Rozen SG (2014). Mutation signatures of carcinogen exposure: Genome-wide detection and new opportunities for cancer prevention. Genome Med..

[4] Huang MN (2017). Genome-scale mutational signatures of aflatoxin in cells, mice, and human tumors. Genome Res..

[5] Nik-Zainal S (2012). Mutational processes molding the genomes of 21 breast cancers. Cell.

[6] Walker BA (2015). APOBEC family mutational signatures are associated with poor prognosis translocations in multiple myeloma. Nat. Commun..

[7] Burns MB (2013). APOBEC3B is an enzymatic source of mutation in breast cancer. Nature.

[8] Burns MB, Temiz NA, Harris RS (2013). Evidence for APOBEC3B mutagenesis in multiple human cancers. Nat. Genet..

[9] Roberts SA (2013). An APOBEC cytidine deaminase mutagenesis pattern is widespread in human cancers. Nat. Genet..

[10] Davies H (2017). HRDetect is a predictor of BRCA1 and BRCA2 deficiency based on mutational signatures. Nat. Med..

[11] Boot, A. *et al.* Recurrent mutations in topoisomerase IIα cause a novel mutator phenotype in human cancers. (In revision). 10.1073/pnas.2114024119 PMC8795545 35058360

[12] Alexandrov LB (2015). Clock-like mutational processes in human somatic cells. Nat. Genet..

[13] Martincorena I (2018). Somatic mutant clones colonize the human esophagus with age. Science.

[14] Martincorena I (2015). Tumor evolution. High burden and pervasive positive selection of somatic mutations in normal human skin. Science.

[15] Li R (2020). Macroscopic somatic clonal expansion in morphologically normal human urothelium. Science.

[16] Lee-Six H (2019). The landscape of somatic mutation in normal colorectal epithelial cells. Nature.

[17] Brunner SF (2019). Somatic mutations and clonal dynamics in healthy and cirrhotic human liver. Nature.

[18] Lawson ARJ (2020). Extensive heterogeneity in somatic mutation and selection in the human bladder. Science.

[19] Yoshida K (2020). Tobacco smoking and somatic mutations in human bronchial epithelium. Nature.

[20] Polak P (2017). A mutational signature reveals alterations underlying deficient homologous recombination repair in breast cancer. Nat. Genet..

[21] Ng AWT (2017). Aristolochic acids and their derivatives are widely implicated in liver cancers in Taiwan and throughout Asia. Sci. Transl. Med..

[22] Kucab JE (2020). A compendium of mutational signatures of environmental agents. Cell.

[23] Boot A (2018). In-depth characterization of the cisplatin mutational signature in human cell lines and in esophageal and liver tumors. Genome Res..

[24] Lu Z-N (2020). The mutational features of aristolochic acid-induced mouse and human liver cancers. Hepatology.

[25] Alexandrov LB, Nik-Zainal S, Wedge DC, Campbell PJ, Stratton MR (2013). Deciphering signatures of mutational processes operative in human cancer. Cell Rep..

[26] Roberts, N. D. *Patterns of somatic genome rearrangement in human cancer* PhD thesis, University of Cambridge (2018). 10.17863/CAM.22674.

[27] Gori, K. & Baez-Ortega, A. sigfit: flexible Bayesian inference of mutational signatures. *bioRxiv * (2020). 10.1101/372896.

[28] Blokzijl F, Janssen R, van Boxtel R, Cuppen E (2018). MutationalPatterns: comprehensive genome-wide analysis of mutational processes. Genome Medicine.

[29] Huang X, Wojtowicz D, Przytycka TM (2018). Detecting presence of mutational signatures in cancer with confidence. Bioinformatics.

[30] Omichessan H, Severi G, Perduca V (2019). Computational tools to detect signatures of mutational processes in DNA from tumours: A review and empirical comparison of performance. PLoS ONE.

[31] Islam, S. M. A. *et al.* Uncovering novel mutational signatures by de novo extraction with SigProfilerExtractor. *bioRxiv*, 2020.2012.2013.422570. 10.1101/2020.12.13.422570 (2021). 10.1016/j.xgen.2022.100179 PMC9646490 36388765

[32] Ardin M (2016). MutSpec: a Galaxy toolbox for streamlined analyses of somatic mutation spectra in human and mouse cancer genomes. BMC Bioinformatics.

[33] Funnell T (2019). Integrated structural variation and point mutation signatures in cancer genomes using correlated topic models. PLOS Computat. Biol..

[34] Fischer A, Illingworth CJR, Campbell PJ, Mustonen V (2013). EMu: probabilistic inference of mutational processes and their localization in the cancer genome. Genome Biol..

[35] Carlson J, Li JZ, Zöllner S (2018). Helmsman: fast and efficient mutation signature analysis for massive sequencing datasets. BMC Genomics.

[36] Mayakonda A, Lin D-C, Assenov Y, Plass C, Koeffler HP (2018). Maftools: Efficient and comprehensive analysis of somatic variants in cancer. Genome Res..

[37] Rosales RA, Drummond RD, Valieris R, Dias-Neto E, da Silva IT (2020). signeR: an empirical Bayesian approach to mutational signature discovery. Bioinformatics.

[38] Robinson W, Sharan R, Leiserson MDM (2019). Modeling clinical and molecular covariates of mutational process activity in cancer. Bioinformatics.

[39] Wang S (2021). Copy number signature analysis tool and its application in prostate cancer reveals distinct mutational processes and clinical outcomes. PLoS Genet..

[40] Degasperi A (2020). A practical framework and online tool for mutational signature analyses show intertissue variation and driver dependencies. Nat. Cancer.

[41] Gehring JS, Fischer B, Lawrence M, Huber W (2015). SomaticSignatures: inferring mutational signatures from single-nucleotide variants. Bioinformatics.

[42] Fantini D, Vidimar V, Yu Y, Condello S, Meeks JJ (2020). MutSignatures: an R package for extraction and analysis of cancer mutational signatures. Sci. Rep..

[43] Lal, A., Liu, K., Tibshirani, R., Sidow, A. & Ramazzotti, D. De Novo Mutational Signature Discovery in Tumor Genomes using SparseSignatures. *bioRxiv*, 384834. 10.1101/384834 (2020). 10.1371/journal.pcbi.1009119 PMC8270462 34181655

[44] Brunet J-P, Tamayo P, Golub TR, Mesirov JP (2004). Metagenes and molecular pattern discovery using matrix factorization. Proc. Natl. Acad. Sci..

[45] Gaujoux R, Seoighe C (2010). A flexible R package for nonnegative matrix factorization. BMC Bioinformatics.

