# Supplementary material for: Accuracy of mutational signature software on correlated signatures
Source: Sci Rep. 2022 Jan 10;12:390. doi: 10.1038/s41598-021-04207-6 (PMC8748538; doi:10.1038/s41598-021-04207-6)
Supplement: Supplementary file 1 — Supplementary Figures. [file 41598_2021_4207_MOESM1_ESM.pdf]

Accuracy of Mutational Signature Software on Correlated Signatures

Yang Wu, Ellora Hui Zhen Chua, Alvin Wei Tian Ng, Arnoud Boot, Steven G. Rozen

Supplementary Figures

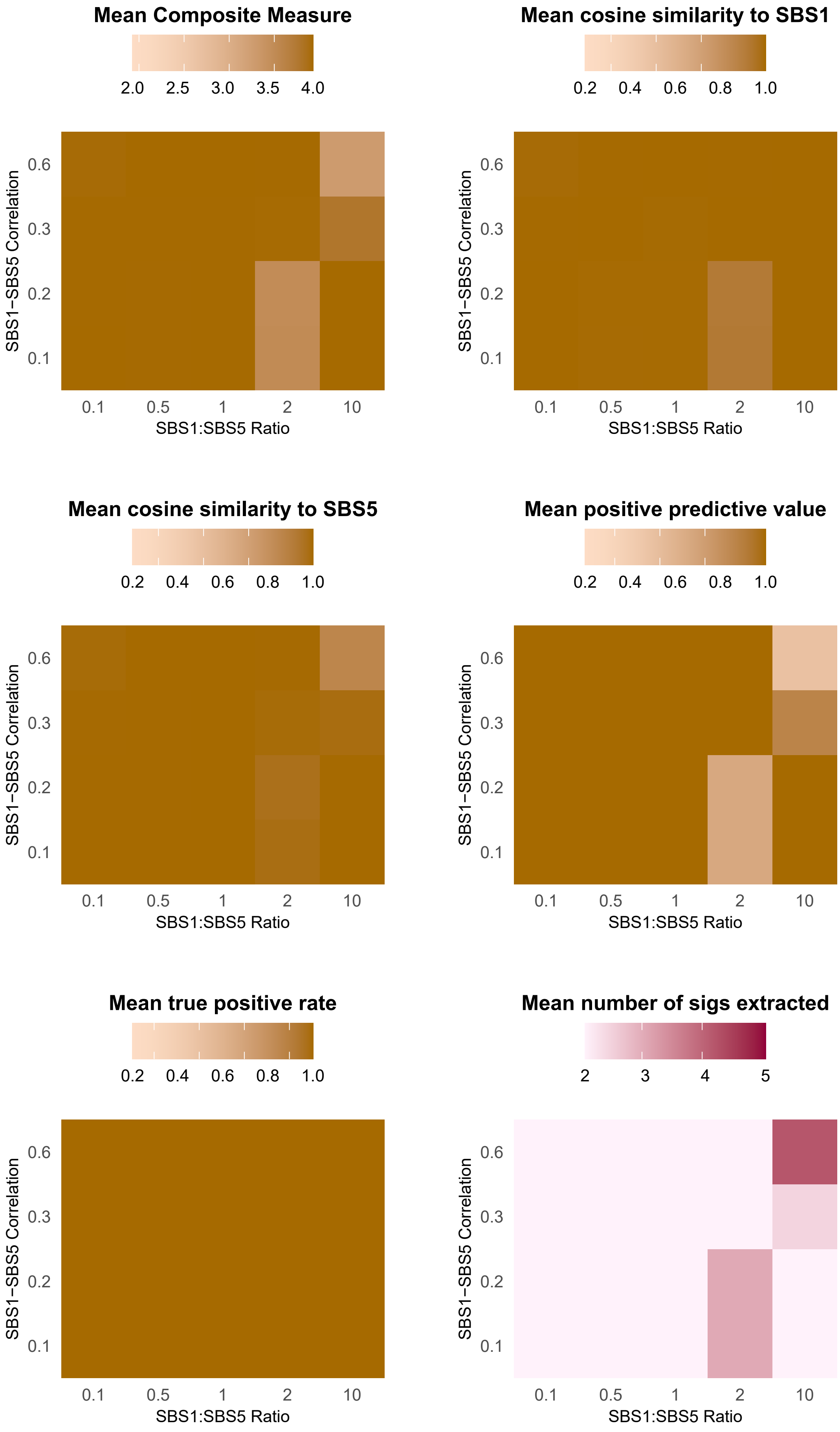

Supplementary Figure S1 Mean evaluation measures and mean number of signatures extracted for hdp when K was unspecified, as a function of SBS1:SBS5 Ratio and SBS1-SBS5 Correlation.

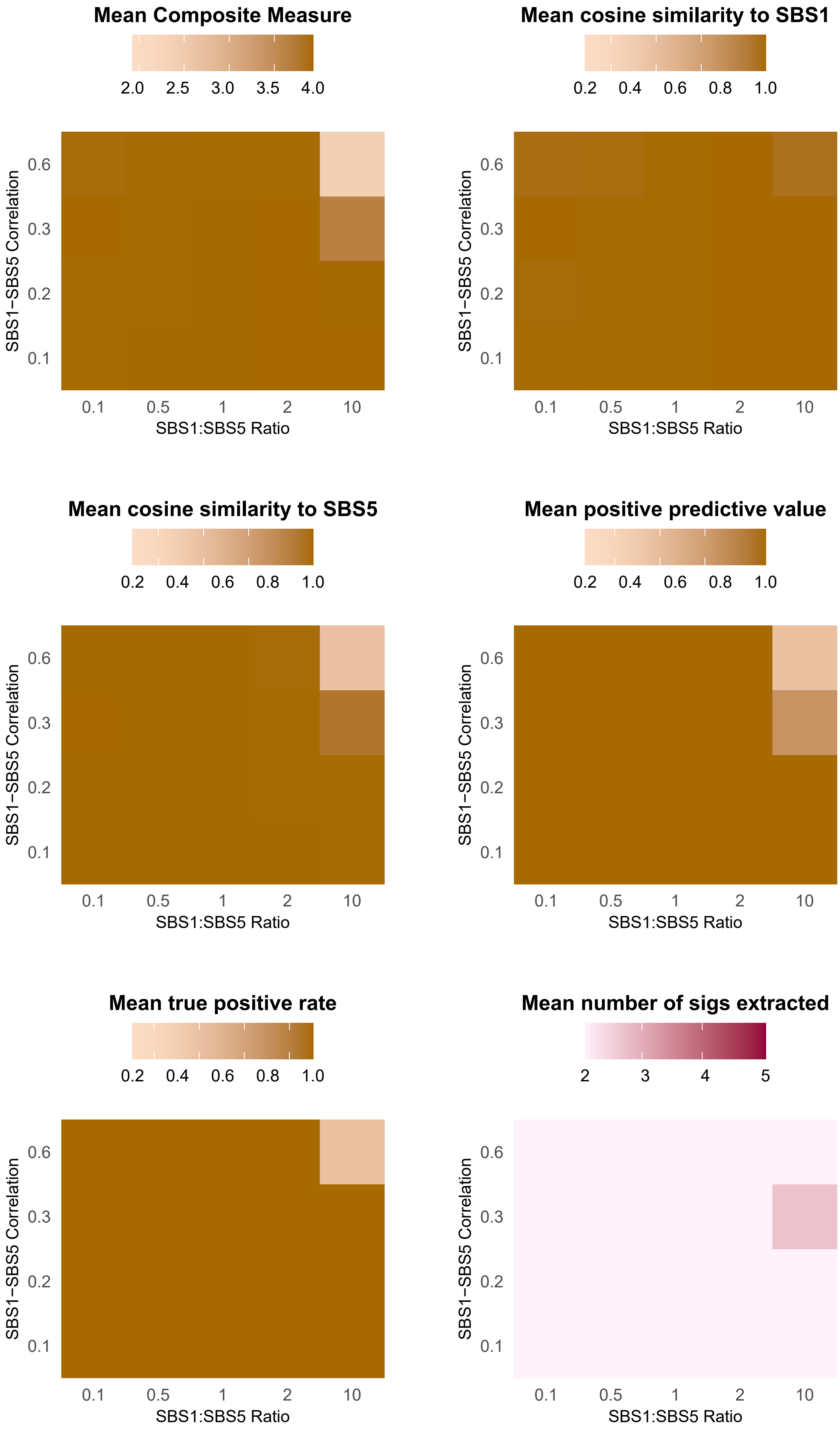

Supplementary Figure S2 Mean evaluation measures and mean number of signatures extracted for SigProExtractor when K was unspecified, as a function of SBS1:SBS5 Ratio and SBS1-SBS5 Correlation.

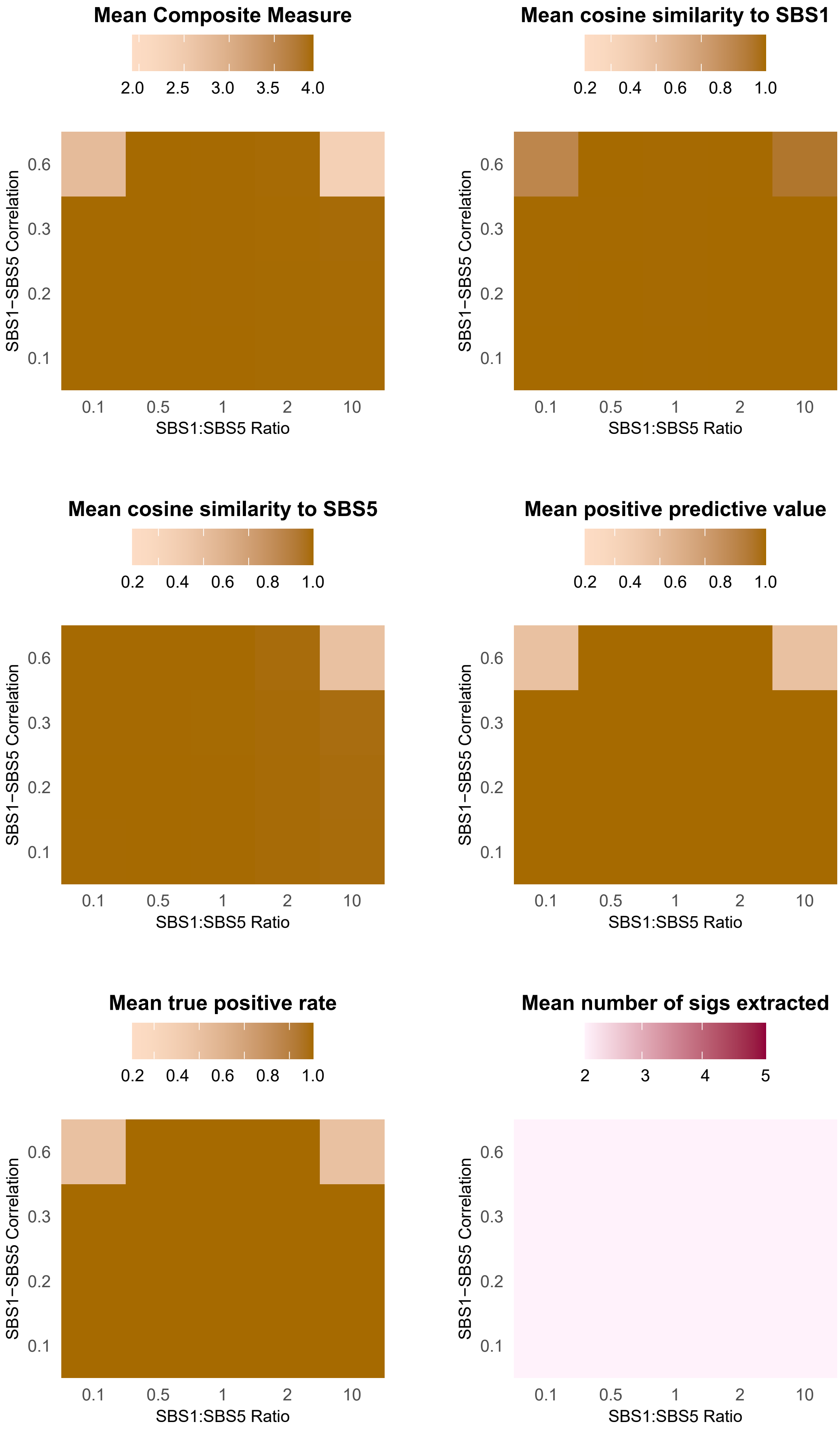

Supplementary Figure S3 Mean evaluation measures and mean number of signatures extracted for TCSM when K was unspecified, as a function of SBS1:SBS5 Ratio and SBS1-SBS5 Correlation.

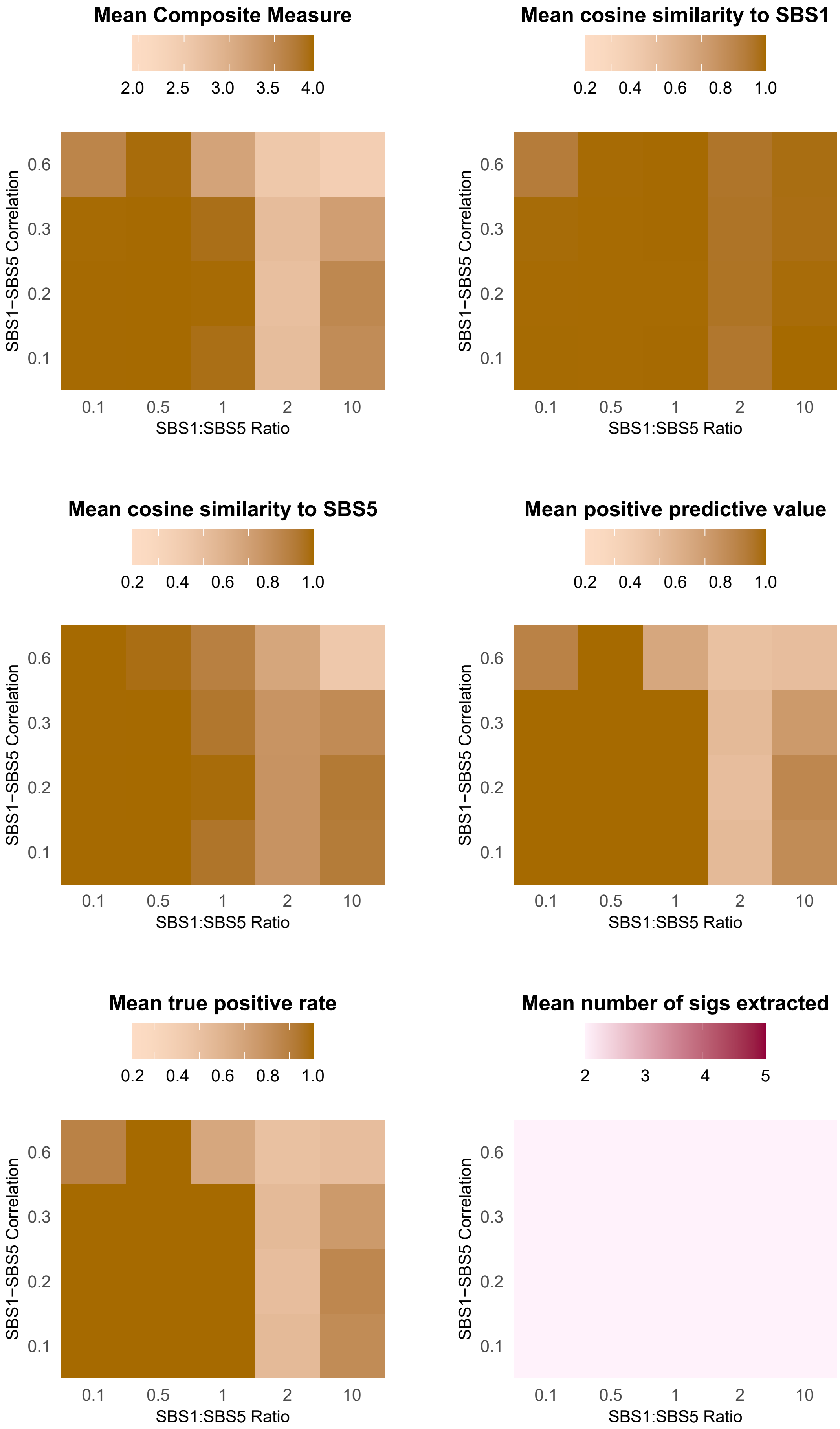

Supplementary Figure S4 Mean evaluation measures and mean number of signatures extracted for mutSpec.NMF when K was unspecified, as a function of SBS1:SBS5 Ratio and SBS1-SBS5 Correlation.

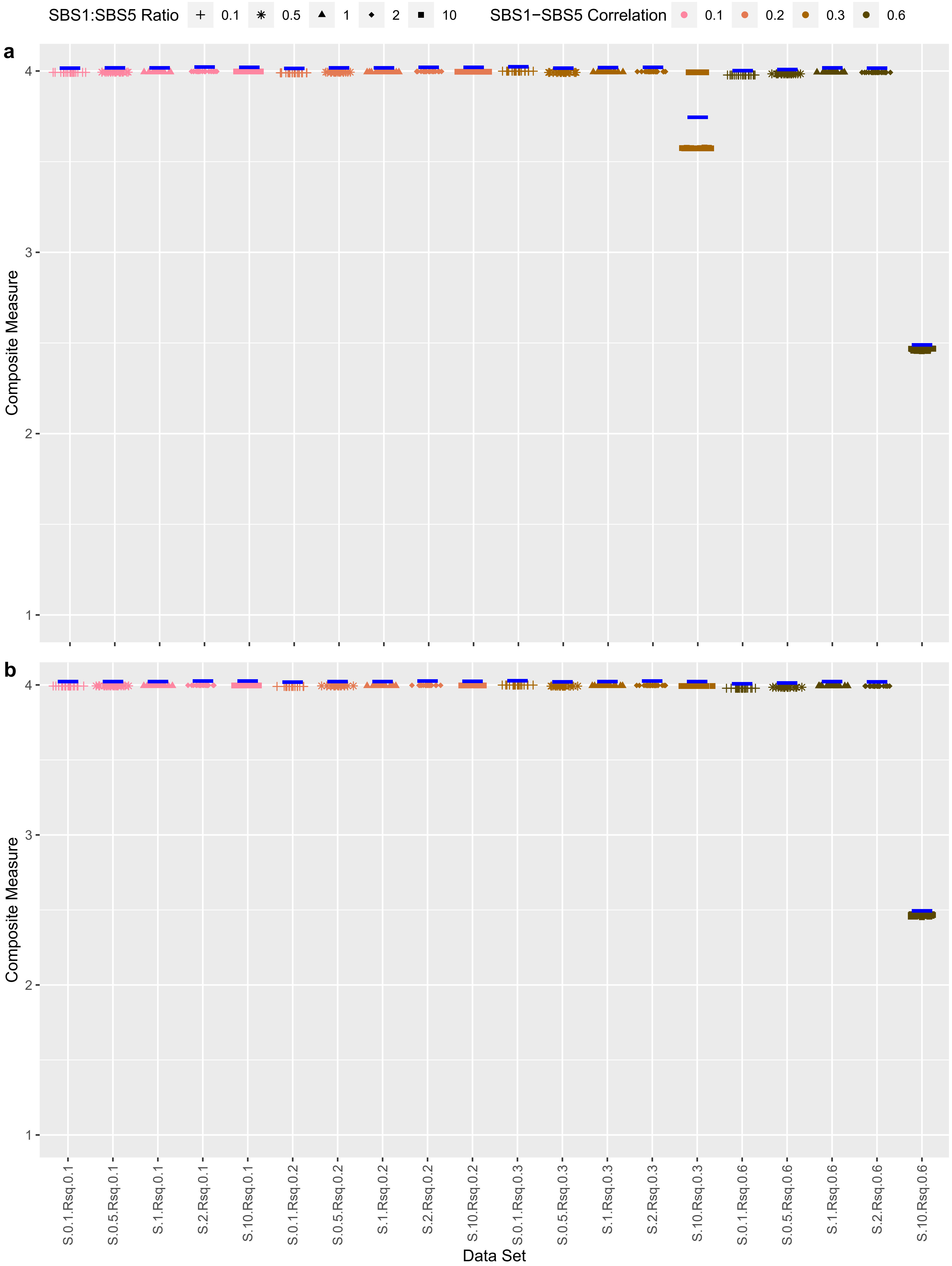

Supplementary Figure S5 Stability of Composite Measures for SigProExtractor for each data set. Each column shows Composite Measures over one data set for all random seeds. Blue bars indicate mean Composite Measures. **(a)** K unspecified; **(b)** K specified as 2.

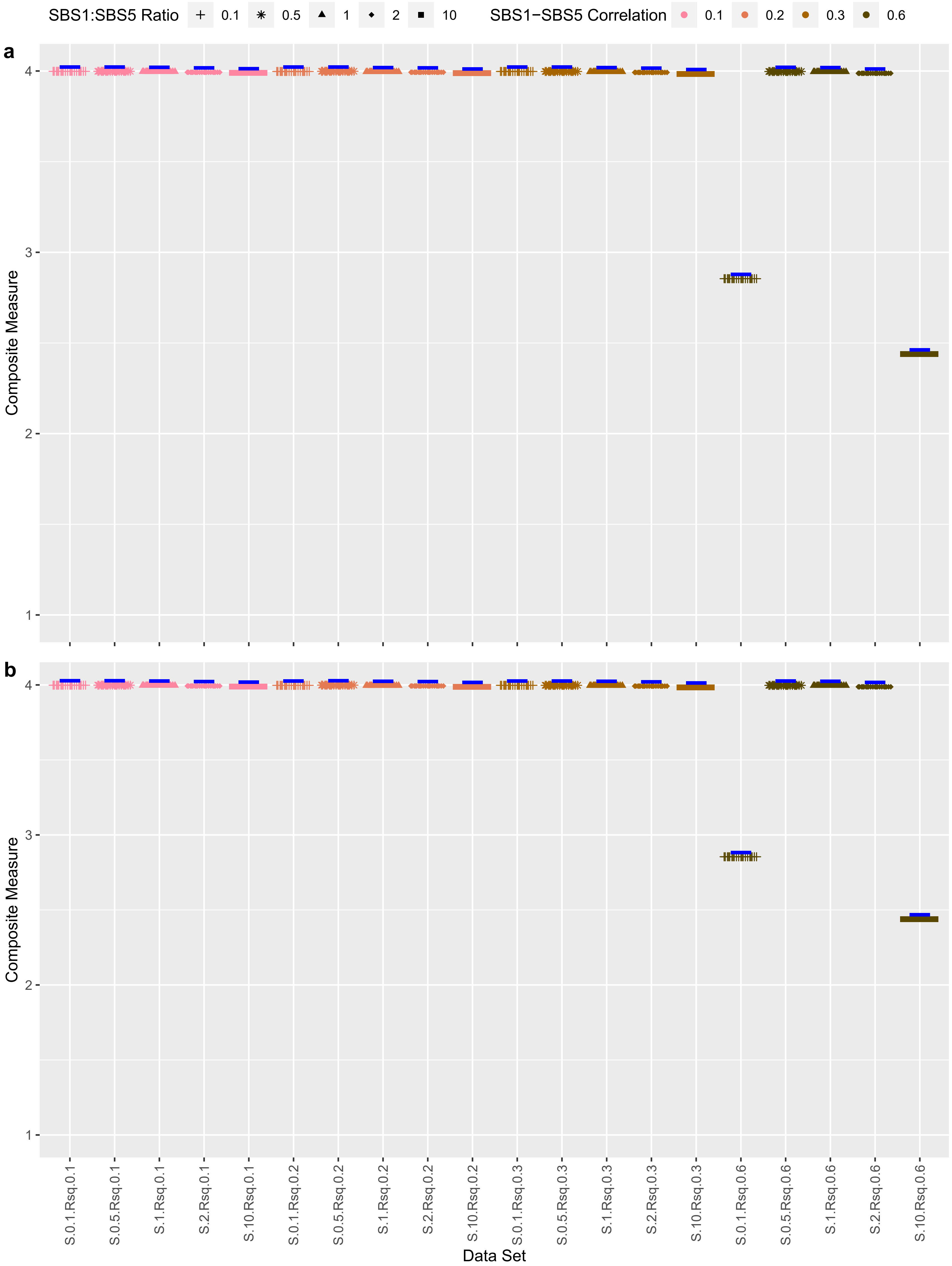

Supplementary Figure S6 Stability of Composite Measures for TCSM for each data set. Each column shows Composite Measures over one data set for all random seeds. Blue bars indicate mean Composite Measures. **(a)** K unspecified; **(b)** K specified as 2.

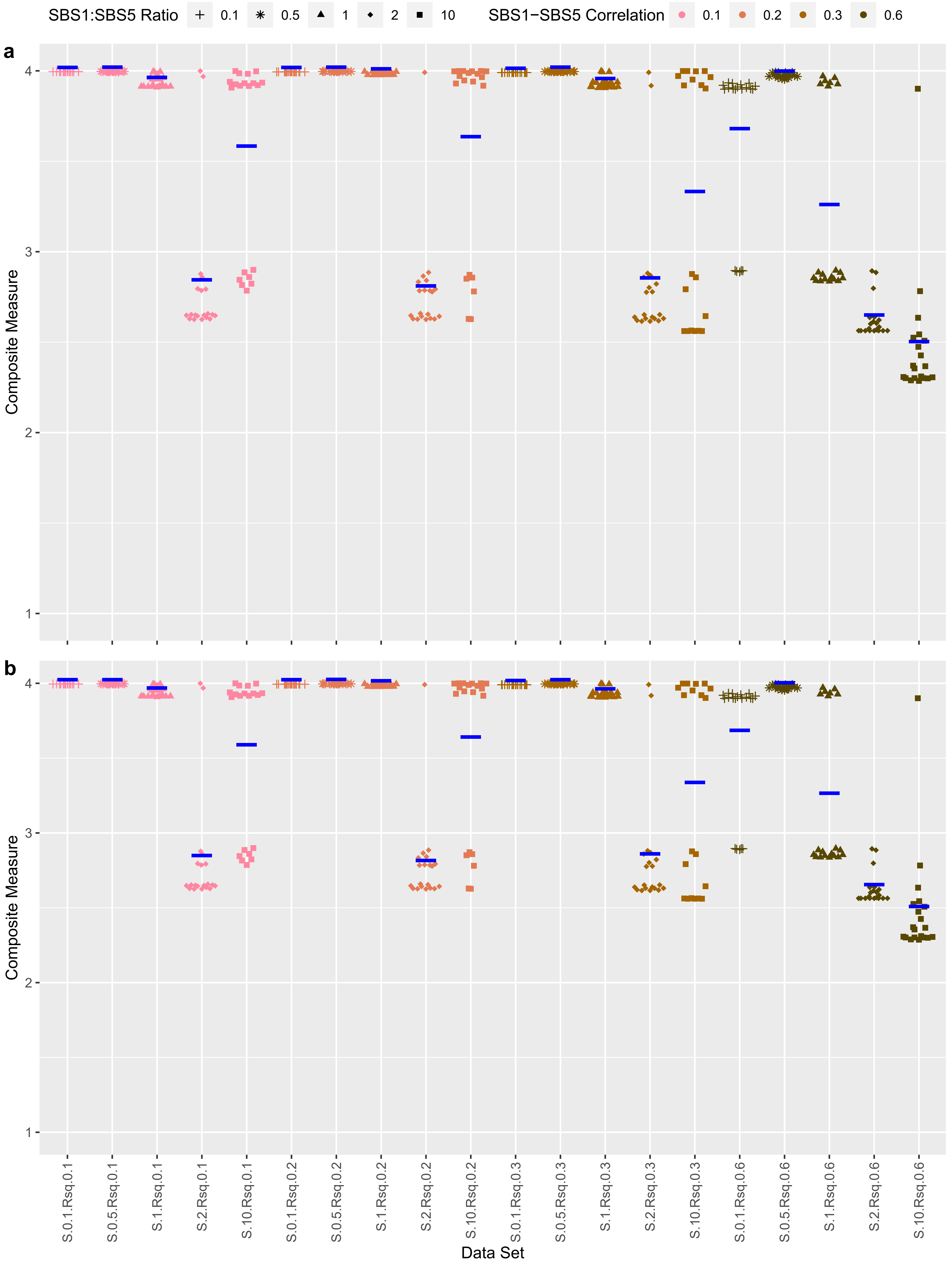

Supplementary Figure S7 Stability of Composite Measures for mutSpec.NMF for each data set. Each column shows Composite Measures over one data set for all random seeds. Blue bars indicate mean Composite Measures. **(a)** K unspecified; **(b)** K specified as 2.

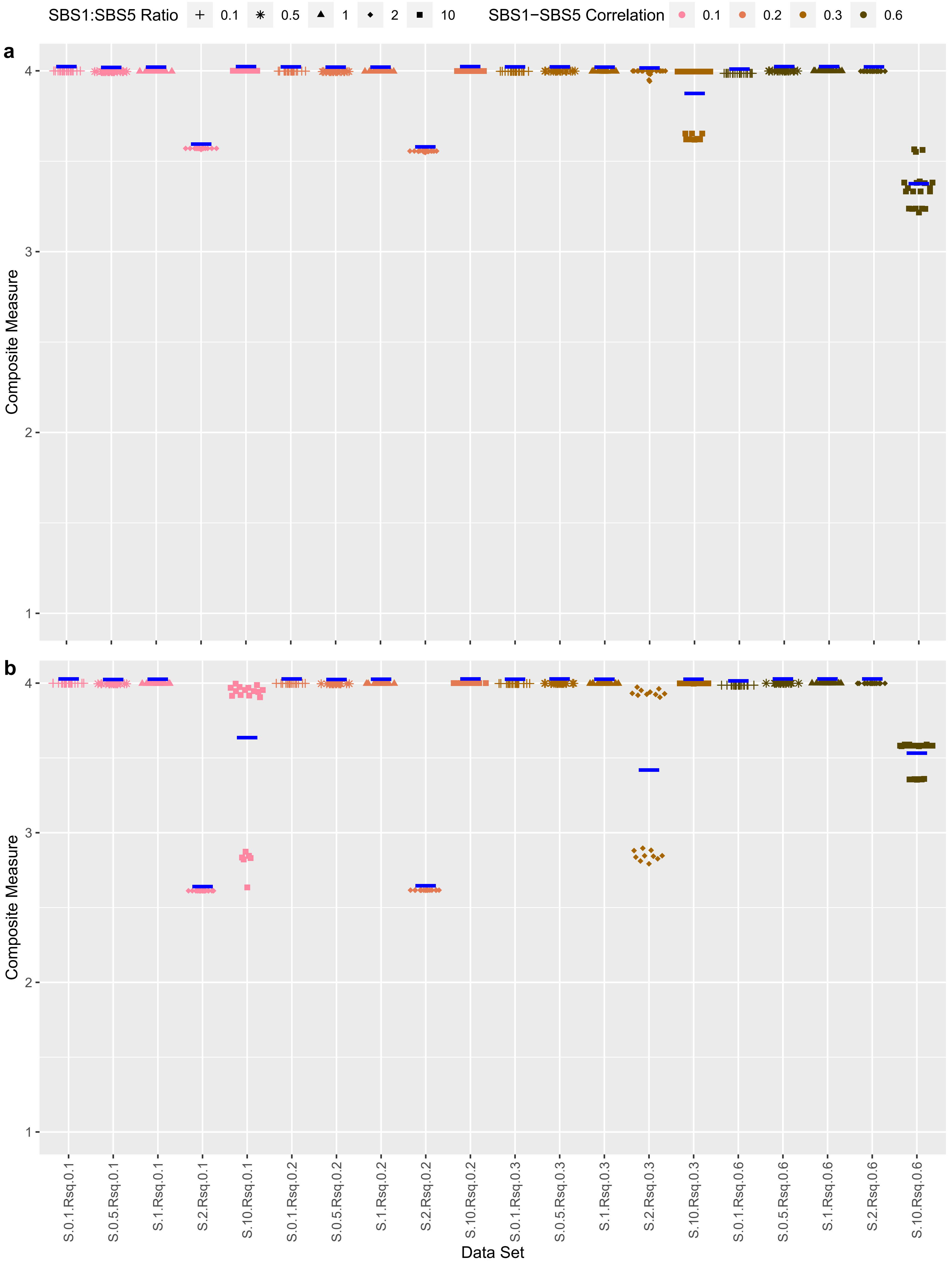

Supplementary Figure S8 Stability of Composite Measures for hdp for each data set. Each column shows Composite Measures over one data set for all random seeds. Blue bars indicate mean Composite Measures. **(a)** K unspecified; **(b)** K specified as 2.

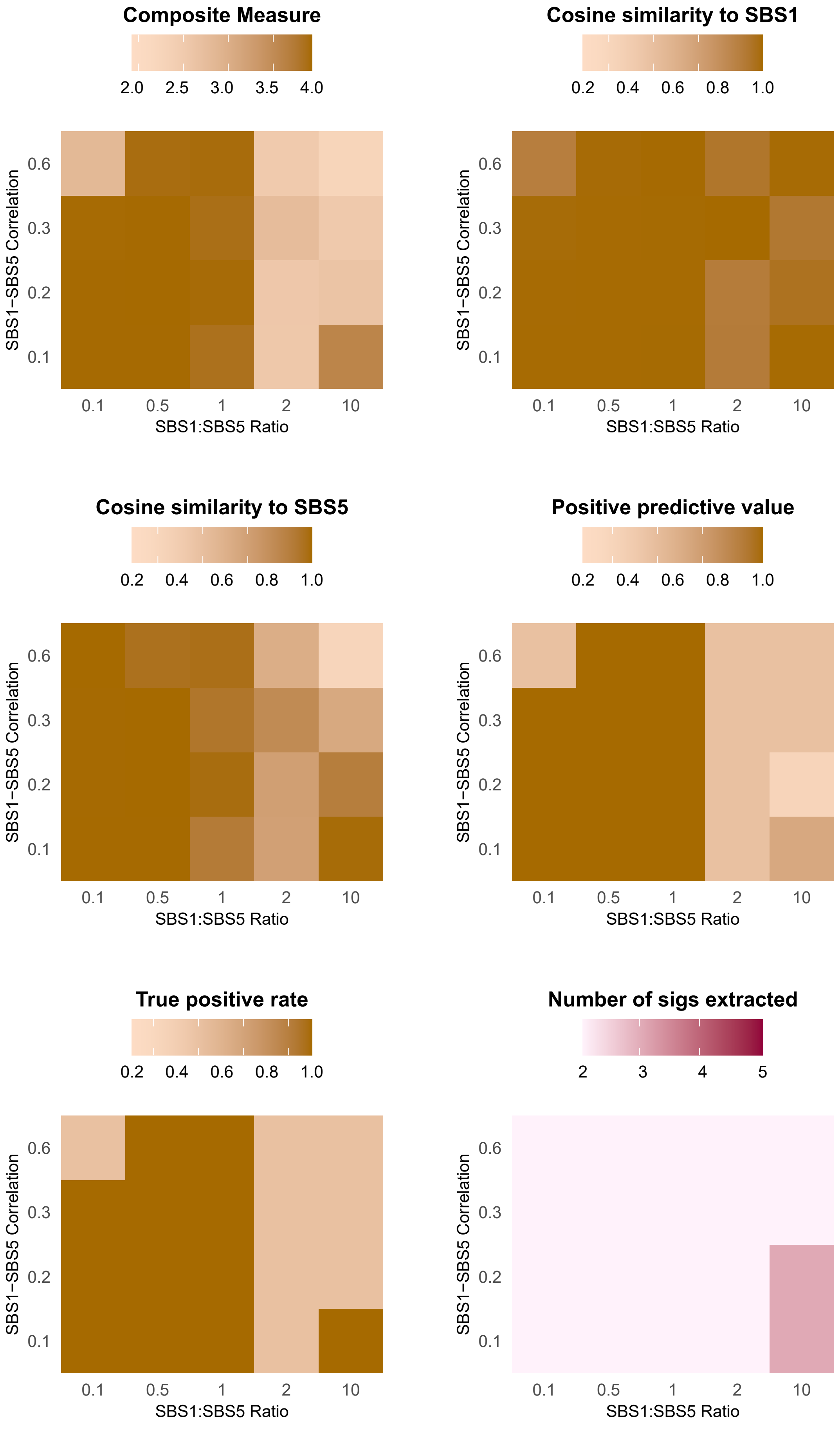

Supplementary Figure S9 Evaluation measures for MutationalPatterns and number of signatures extracted when K was unspecified, as a function of SBS1:SBS5 Ratio and SBS1-SBS5 Correlation. Mean was not calculated, as there was only one run with hard-coded seed for each data set.

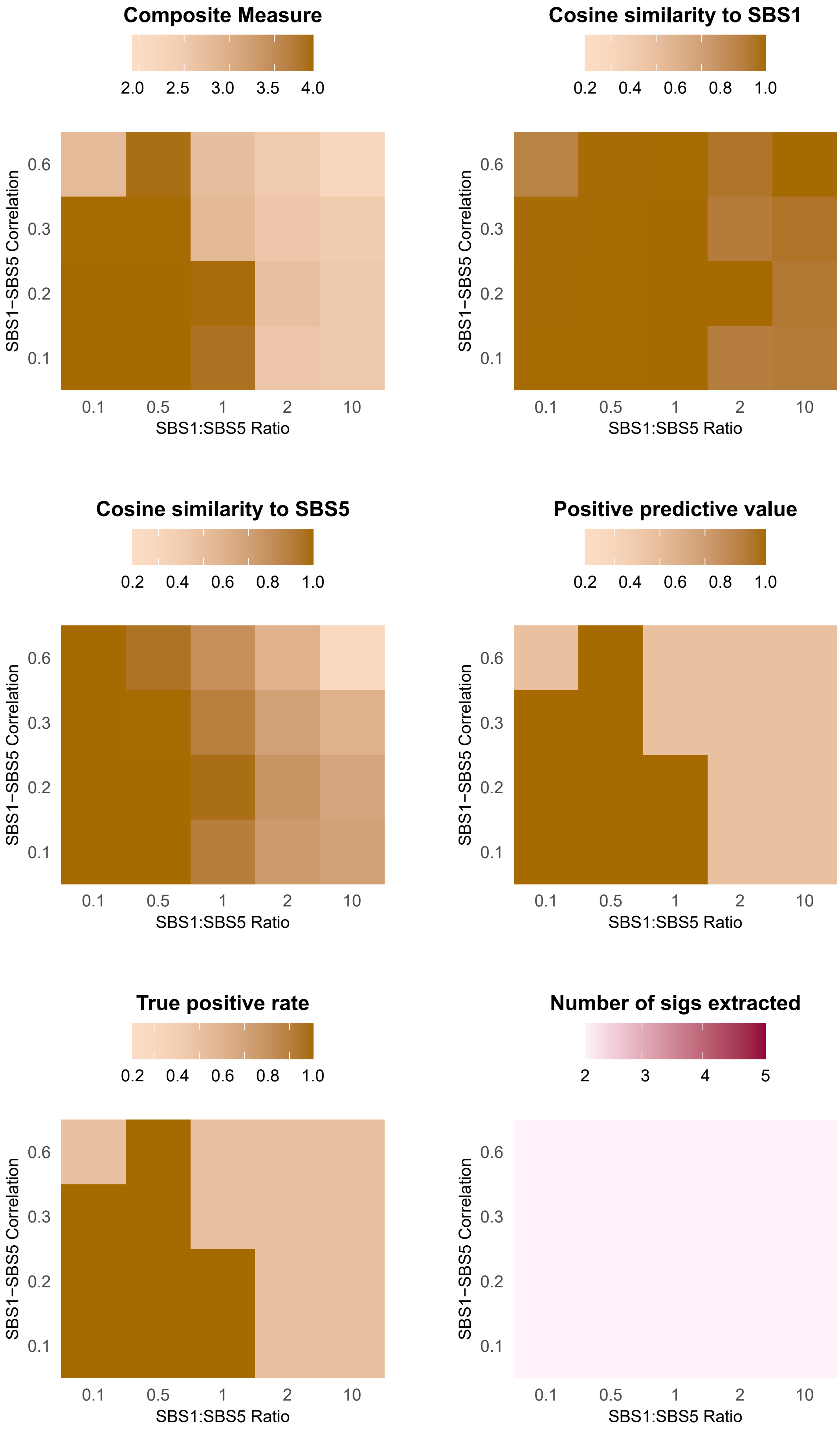

Supplementary Figure S10 Evaluation measures for maftools and number of signatures extracted, as a function of SBS1:SBS5 Ratio and SBS1-SBS5 Correlation. Mean was not calculated, as there was only one run with hard-coded seed for each data set.

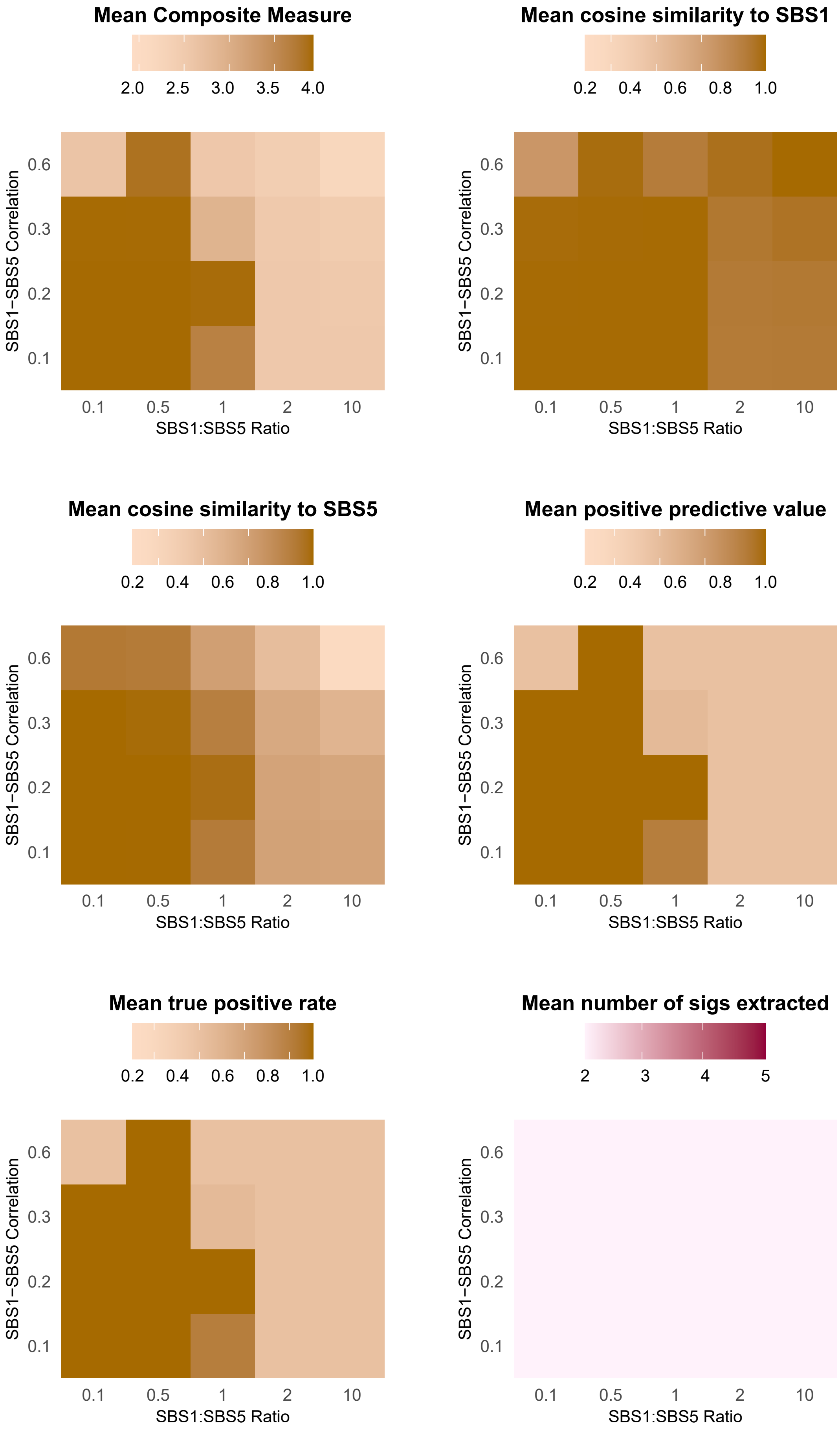

Supplementary Figure S11 Mean evaluation measures and mean number of signatures extracted for signeR when K was unspecified, as a function of SBS1:SBS5 Ratio and SBS1-SBS5 Correlation.

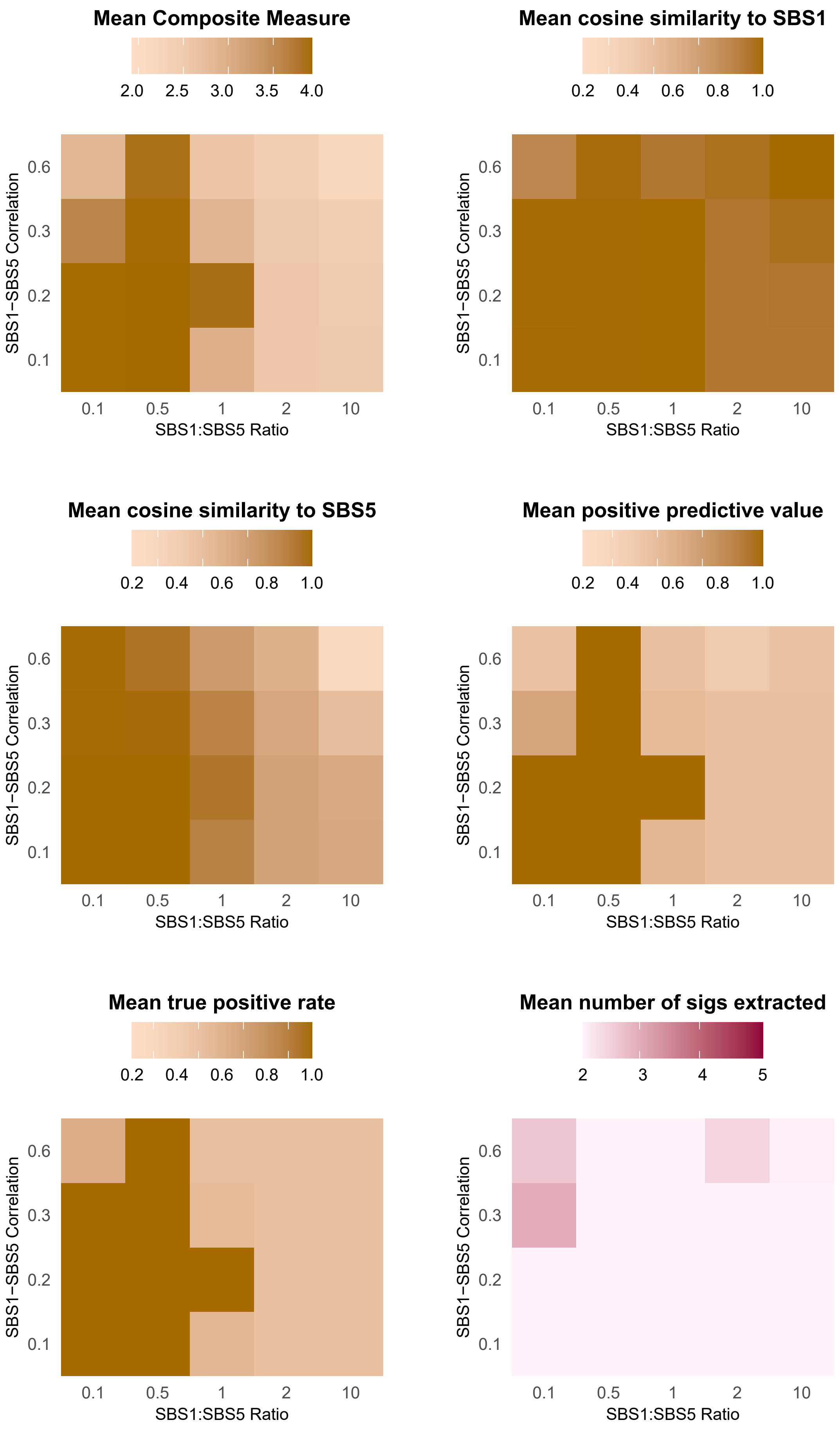

Supplementary Figure S12 Mean evaluation measures and mean number of signatures extracted for helmsman.NMF, when K was unspecified, as a function of SBS1:SBS5 Ratio and SBS1-SBS5 Correlation.

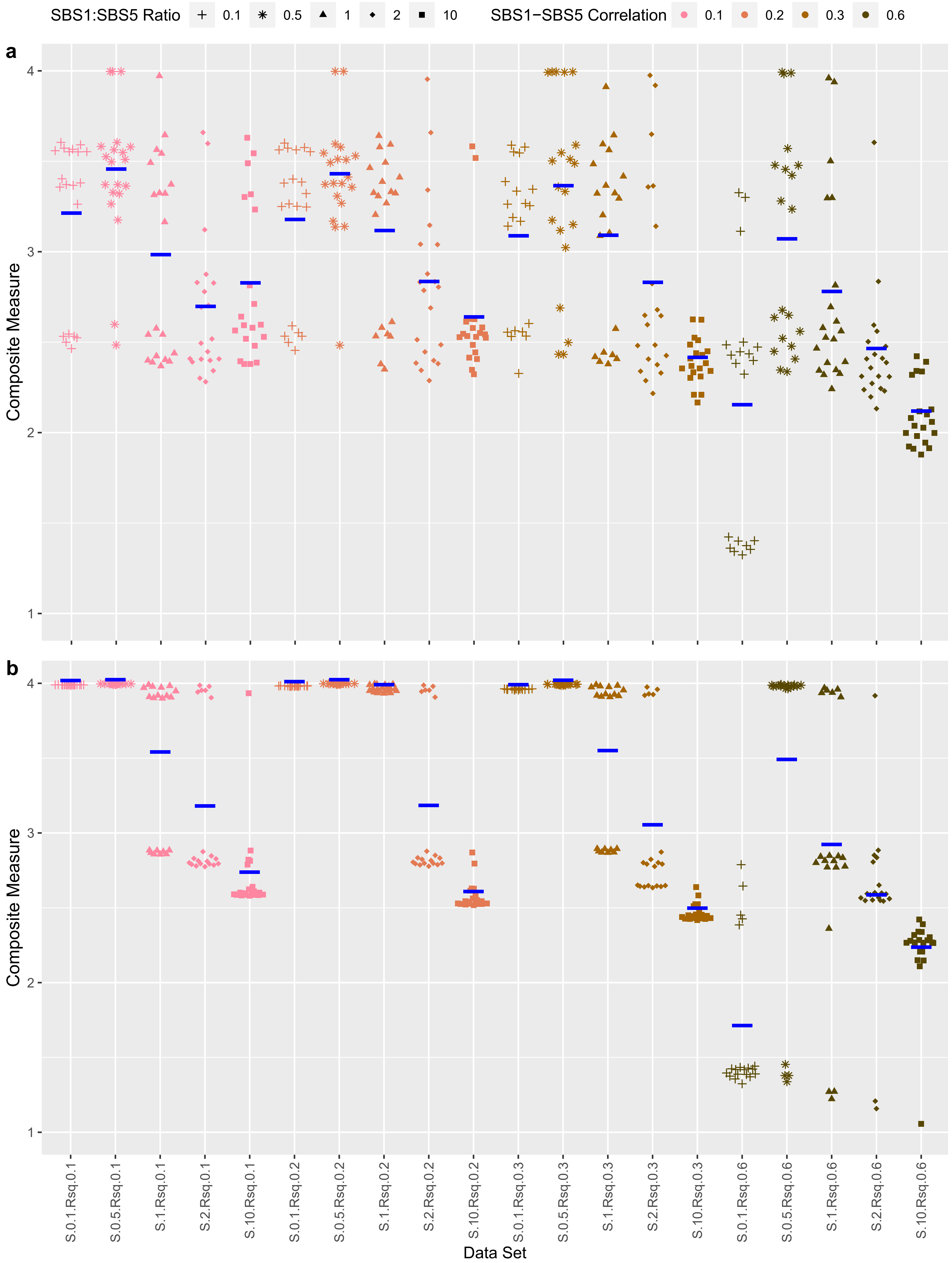

Supplementary Figure S13 Stability of Composite Measures for MultiModalMuSig.LDA for each data set. Each column shows Composite Measures over one data set for all random seeds. Blue bars indicate mean Composite Measures. **(a)** K unspecified; **(b)** K specified as 2.

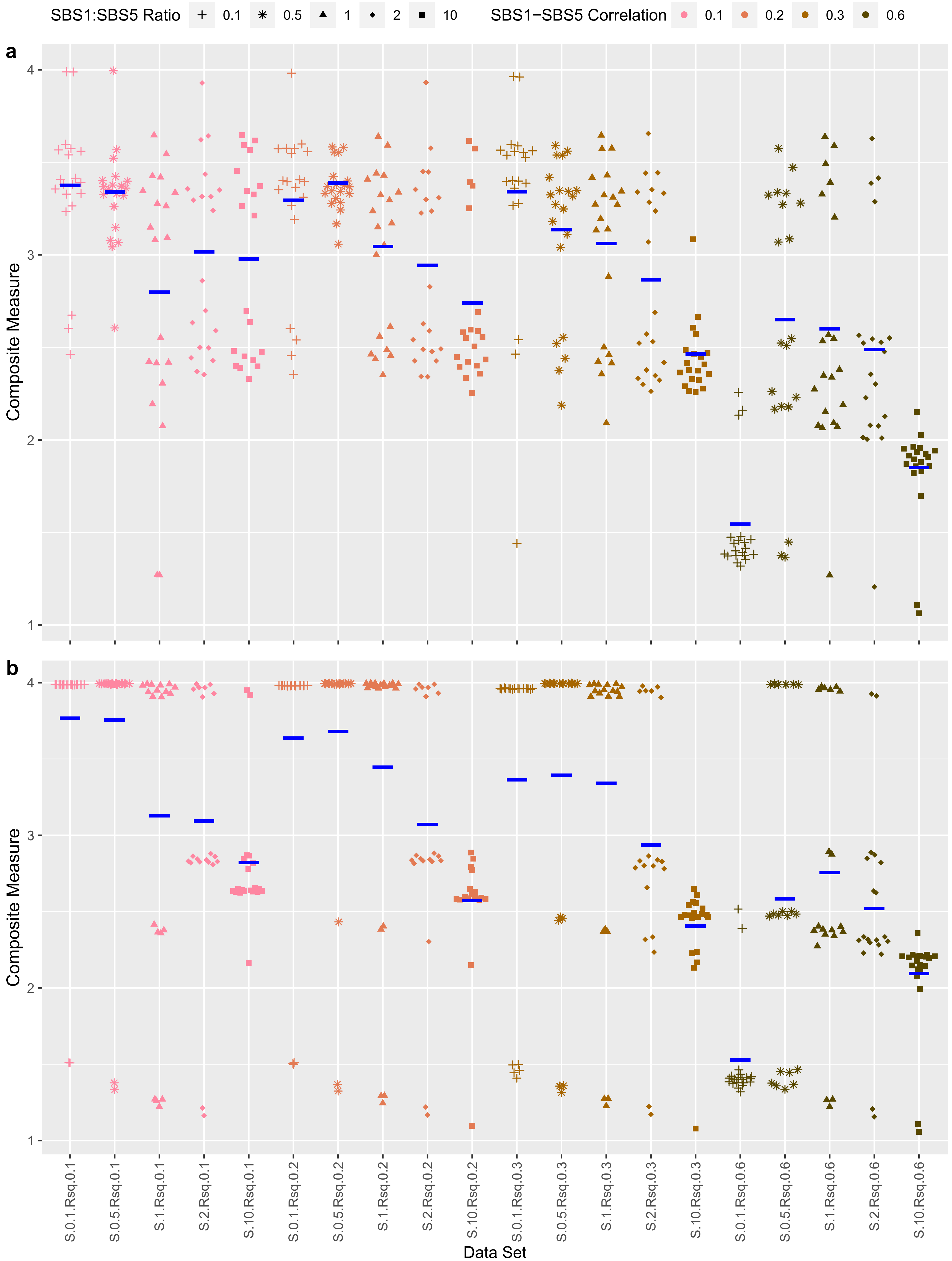

Supplementary Figure S14 Stability of Composite Measures for MultiModalMuSig.MMCTM for each data set. Each column shows Composite Measures over one data set for all random seeds. Blue bars indicate mean Composite Measures. **(a)** K unspecified; **(b)** K specified as 2.

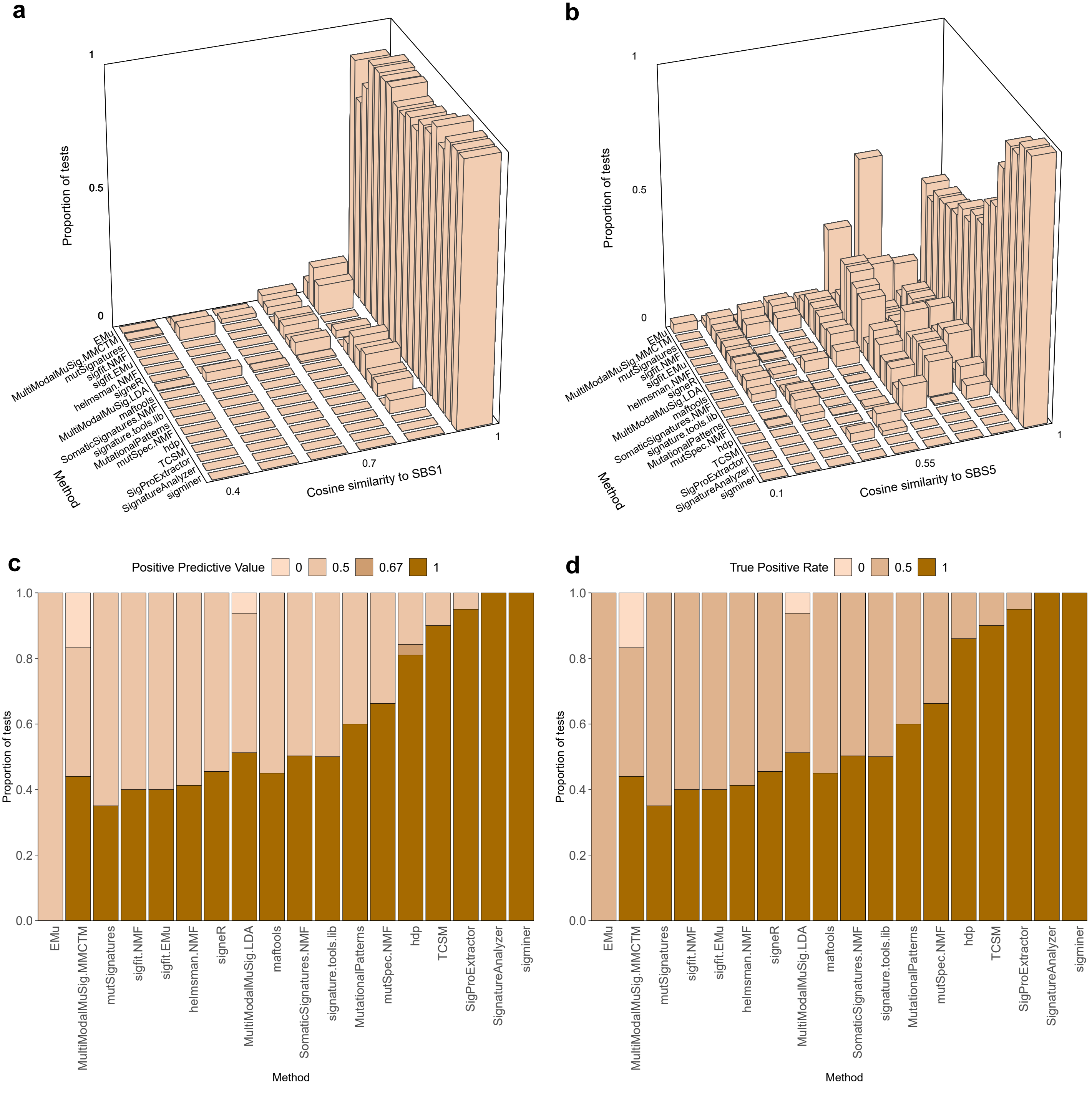

Supplementary Figure S15 Individual measures of signature extraction performance when the number of signatures was specified as 2. The methods are arranged in order of ascending mean Composite Measure from back to front, as in main text Fig. 7.

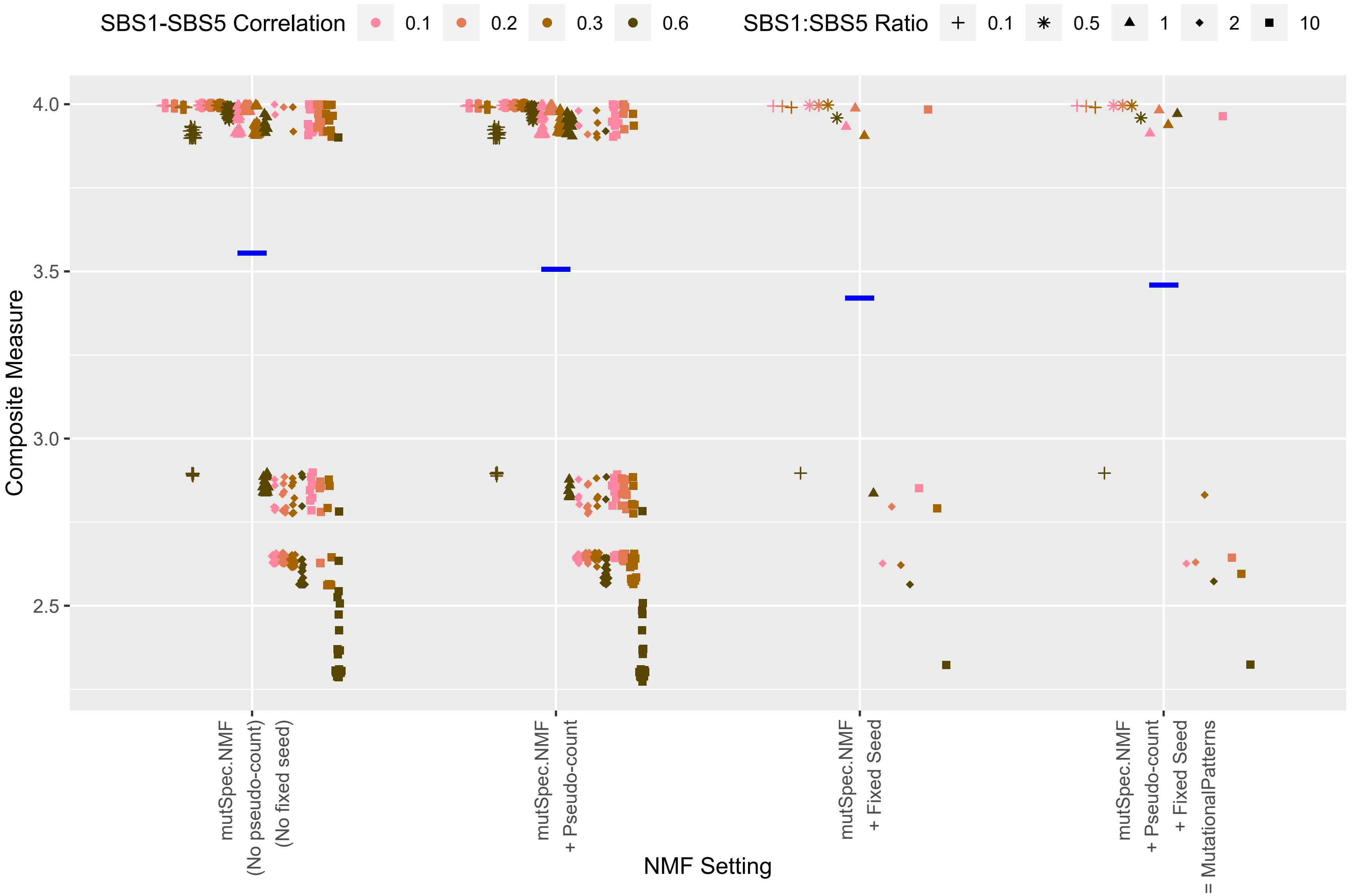

Supplementary Figure S16 The reason that MutationalPatterns has lower Composite Measures than mutSpec.NMF. Each column shows the Composite Measures over all data sets and all random seeds. Data were generated with nrun = 200 and K as 2. Blue bar indicates mean Composite Measure.

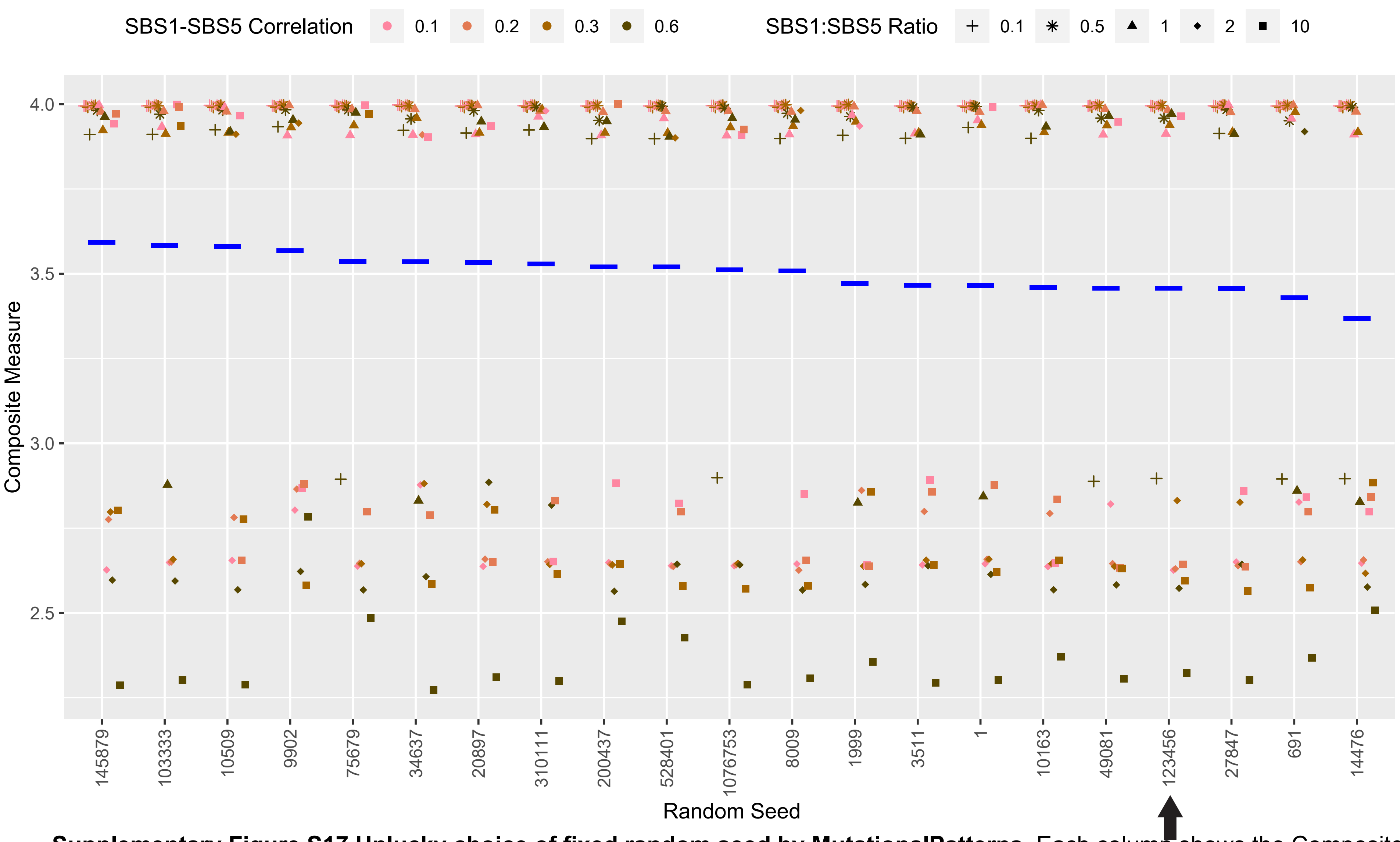

Supplementary Figure S17 Unlucky choice of fixed random seed by MutationalPatterns. Each column shows the Composite Measures over all data sets for a single random seed. Data were generated with nrun = 200 and with pseudocount added, i.e the settings used by MutationalPatterns. The fixed random seed used by MutationalPatterns is indicated with an arrow, and generates a mean Composite Measure, indicated by the blue bar, worse than the average of the mean Composite Measures over all the other random seeds.

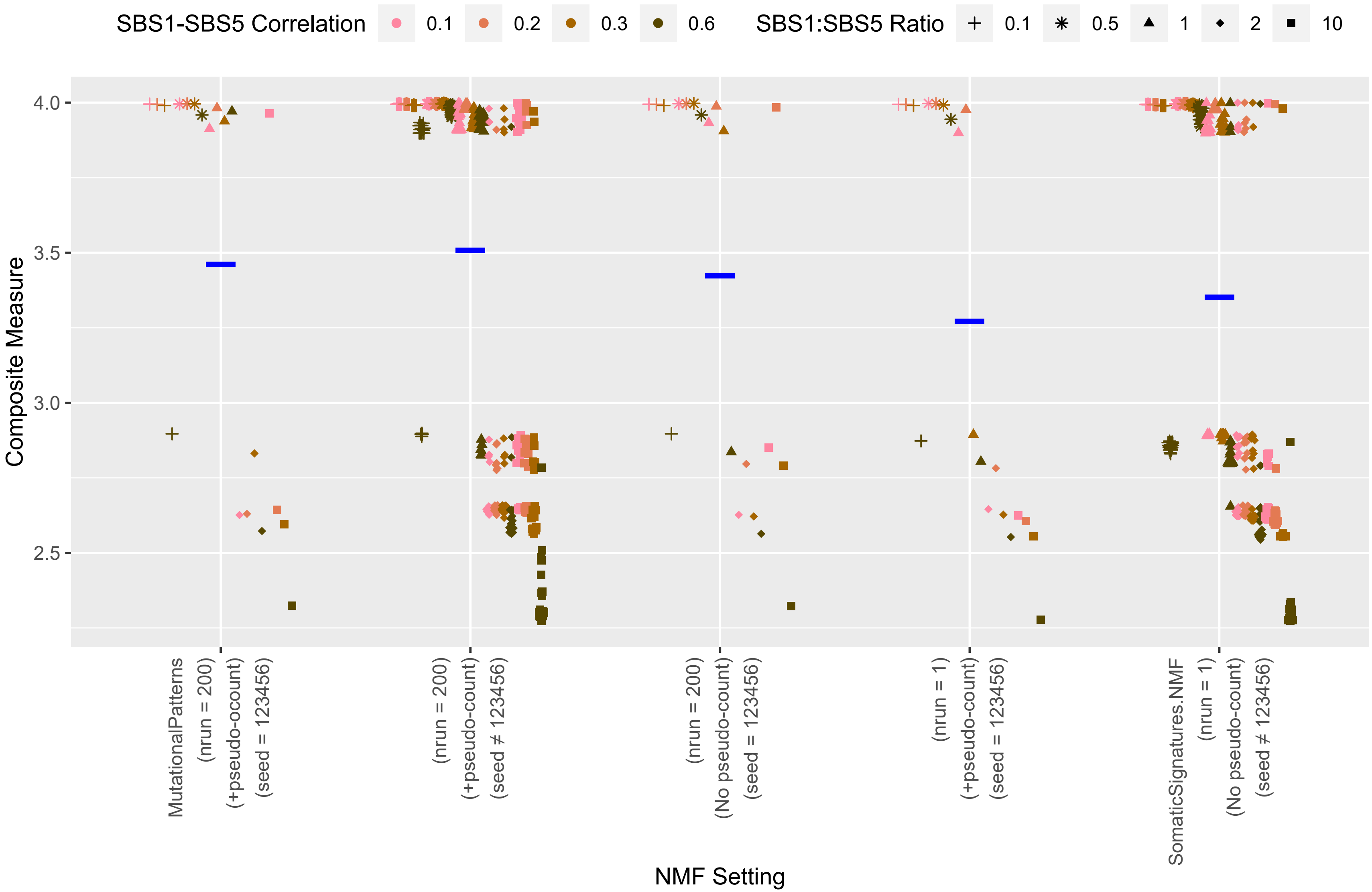

Supplementary Figure S18 The reason that SomaticSignatures.NMF has lower Composite Measures than MutationalPatterns. Each column shows the Composite Measures over all data sets and all random seeds. Blue bar indicates mean Composite Measure.

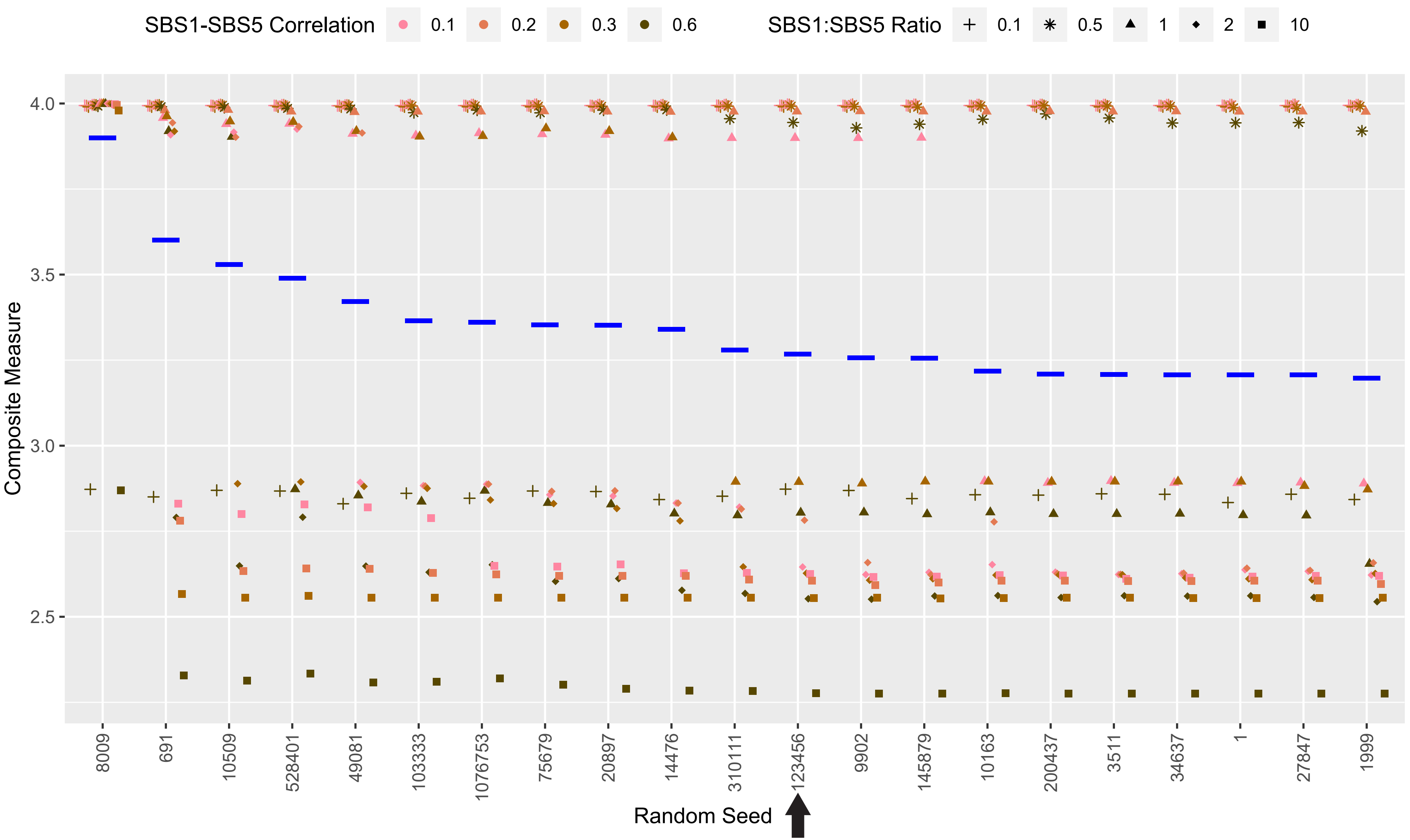

Supplementary Figure S19 The reason that maftools had lower Composite Measure than SomaticSignatures.NMF: unlucky choice of fixed random seed. Each column shows the Composite Measures over all data sets for a single random seed. Data were generated with nrun = 1 and without pseudocount, i.e the settings used by SomaticSignatures.NMF and maftools. The fixed random seed used by maftools is indicated with an arrow, and generates a mean Composite Measure, indicated by the blue bar, worse than the average of the mean Composite Measures over all the other random seeds.

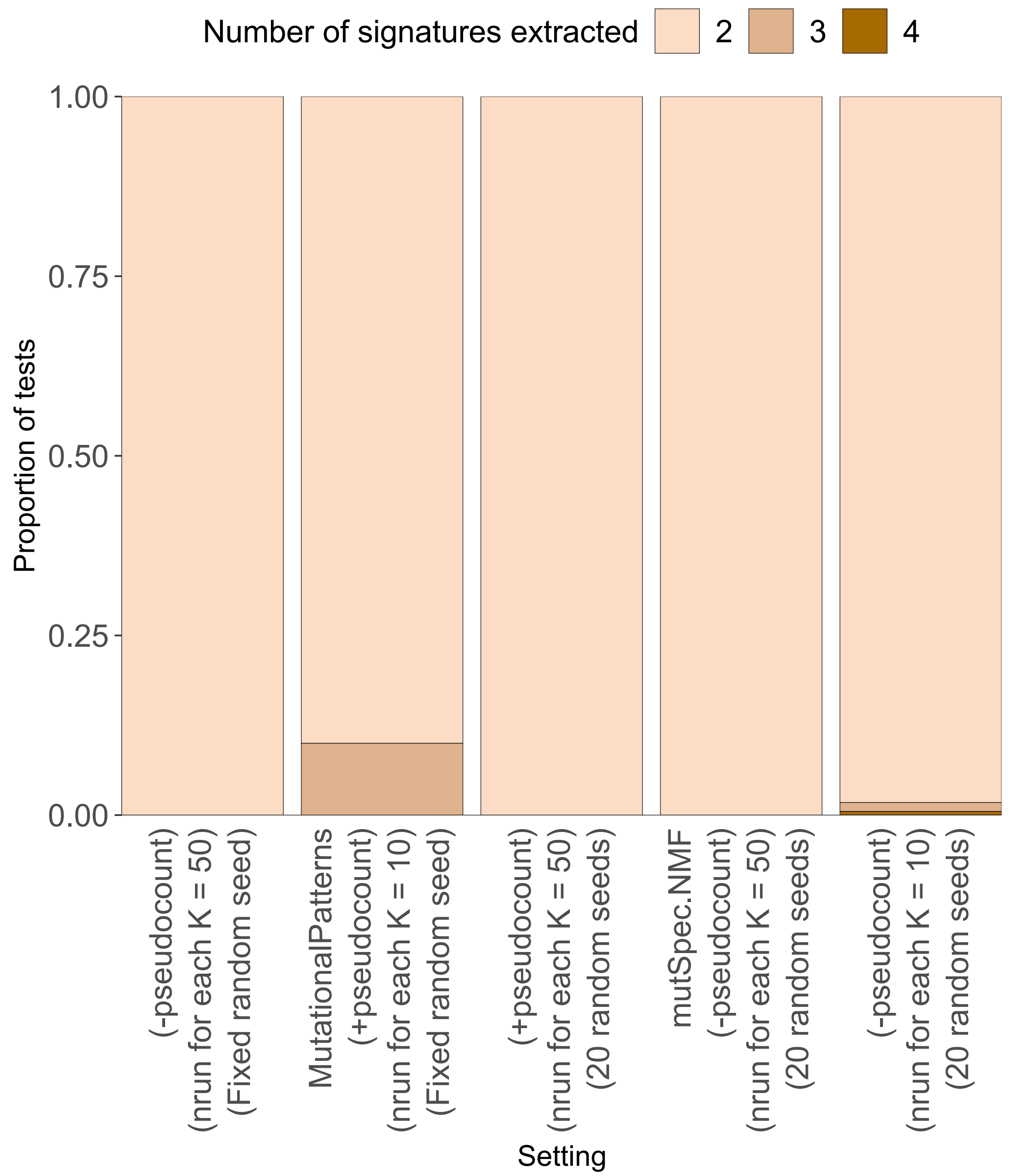

Supplementary Figure S20 Using nrun = 10 in function nmfEstimateRank, MutationalPatterns sometimes extracted > 2 signatures (column 2). The y axis illustrates the proportion of runs over all data sets and random seeds with the number of signatures that were extracted indicated by the color of the bar. Calling nmfEstimateRank with nrun = 50, the value used by mutSpec.NMF, always extracted 2 signatures.
